# Development and initial content validation of the Vienna post-exertional malaise questionnaire: an iterative mixed-methods study

**DOI:** 10.1007/s11136-026-04434-2

**Published:** 2026-11-07

**Authors:** Theresa Pimminger, Annika Ahrens-Schwabe, Tobias Fragner, Tobias Brandl, Stefan T. Kotzian, Rob C.I. Wüst, Thomas Waldhoer, Ali Kapan

**Affiliations:** 1 Center for Public Health, Department of Social and Preventive Medicine, Medical University of Vienna, Kinderspitalgasse 15, Vienna, 1090 Austria; 2 Department of German Studies, University of Vienna, Vienna, Austria; 3 Neurologisches Rehabilitationszentrum Rosenhügel, Vienna, Austria; 4 Department of Human Movement Sciences, Faculty of Behavioural and Movement Sciences, Vrije Universiteit Amsterdam, Amsterdam Movement Sciences, Amsterdam, the Netherlands; 5 Center for Public Health, Department of Epidemiology, Medical University of Vienna, Vienna, Austria

**Keywords:** Post-exertional malaise, Myalgic encephalomyelitis, Chronic fatigue syndrome, Patient-reported outcome measure, Content validity, Instrument development

## Abstract

**Objectives:**

Post-exertional malaise (PEM) is a multidimensional exertion-response pattern involving diverse triggers, variable onset, and prolonged recovery. This study developed and content-validated the Vienna Post-Exertional Malaise (VPEM) Questionnaire, a patient-reported outcome measure assessing functional decline, recovery, crash-associated symptoms, and pacing adaptations in ME/CFS.

**Methods:**

An iterative mixed-methods instrument-development study combined quantitative ratings and qualitative feedback across sequential evaluation cycles. An initial 13-item draft, informed by existing PEM, fatigue, and functional-capacity instruments and consensus literature, was evaluated by individuals with ME/CFS or Post-COVID-19 syndrome (PCS) using Likert ratings, open-ended feedback, and interviews. Quantitative data were summarised descriptively, and qualitative data were analysed using deductive–inductive structuring content analysis. Revisions were documented in revision matrices. The revised instrument was evaluated by an interdisciplinary expert panel using domain-level and item-level ratings of relevance and clarity.

**Results:**

Twelve individuals with ME/CFS and one individual with PCS fulfilling ME/CFS criteria evaluated the questionnaire. Ratings showed high approval for length (92.3%) and terminology (76.9%), while completeness and clarity were more heterogeneous. Qualitative findings identified ambiguities in distinguishing PEM from non-specific fatigue, missing trigger domains, and insufficient representation of intra-individual variability. After revision, the expert panel (*n* = 11) rated PEM coverage as complete or largely complete (100%) and the multidimensional structure as suitable or very suitable (90.9%). Item-level ratings showed high relevance (S-CVI/Ave = 0.96), whereas clarity ratings were more variable (S-CVI/Ave = 0.82).

**Conclusions:**

The VPEM Questionnaire operationalises PEM as multidimensional post-exertional deterioration. Findings support initial content and face validity, warranting further psychometric validation.

**Supplementary Information:**

The online version contains supplementary material available at https://doi.org/10.1007/s11136-026-04434-2.

## Introduction

Myalgic encephalomyelitis/chronic fatigue syndrome (ME/CFS) and Post-COVID-19 syndrome (PCS) are complex and often disabling conditions characterised by post-exertional malaise (PEM), a pathological response to often trivial levels of exertion that involves an abnormal multisystem and multisymptomatic exacerbation.1 This response can lead to substantial functional impairment and, in many cases, severe disability [1, 2]. PEM can be described as a complex, multidimensional and context-dependent phenomenon. It is characterised by a variety of triggers, including physical, cognitive, sensory, emotional or social, and orthostatic demands, as well as variable onset latencies, prolonged and often incomplete recovery, and a broad multisystem symptom profile [3, 4].

PEM has been described as a multidimensional construct, involving variability in triggers, symptom exacerbation, delayed onset, and recovery trajectories. PEM is the worsening of general symptoms, or the development of new symptoms, typically within 48 h after physical, cognitive or mental exertion, above a patient- and time-specific threshold, that can last for days to weeks or even months. Patients experience PEM in a variety of symptoms and severities, but a standard way to capture this in research and clinics is missing. A clearer understanding of whether PEM reflects a primary pathophysiological process or a downstream, system-level manifestation of the primary pathophysiological mechanisms remains an important objective for future research, as its underlying biology is not fully established [5, 6]. A fuller understanding of the pathophysiology of PEM requires robust, standardised measurement approaches that can accommodate the heterogeneity and complexity of PEM [7, 8], but also to link patients symptoms to underlying cellular and molecular pathways, and to better guide future therapeutic interventions. At the same time, validated instruments are needed to systematically assess the clinically observable consequences of PEM, including functional decline, delayed or prolonged recovery, and crash-associated symptom changes. Several instruments have been developed to assess these features [8, 9]. The DePaul Symptom Questionnaire PEM subscale (DSQ-PEM) has demonstrated acceptable reliability and validity in identifying and diagnosing PEM-related symptoms; however, it primarily assesses symptom frequency and severity while providing limited coverage of aspects such as trigger diversity, delayed onset, and recovery dynamics [10]. The newer DSQ-PEM-2 is a shorter alternative addressing some of these limitations, but it remains in an early stage of validation, and the use of initial screening items may influence case identification depending on symptom attribution [11]. The Functional Capacity Scale (FunCap-27) assesses activity-related fatigue and performance decrements across multiple domains. It can indirectly reflect aspects of PEM-related functional impairment, yet it does not explicitly operationalise or quantify the core features of PEM itself [12]. Furthermore, qualitative research suggests that terminology commonly used in existing instruments, such as “fatigue” or “exhaustion”, does not always reflect patients’ lived experience, which is often described using terms such as a “crash” or flu-like deterioration. This mismatch may affect both comprehensibility and content validity [13].

As such, unidimensional or purely symptom-focused assessments do not fully meet current clinical and research needs. Adequate capture of PEM requires a patient-centred, multidimensional instrument that systematically addresses trigger profiles, temporal characteristics, functional impact, associated symptoms, and behavioural adaptations such as pacing [13]. Here, we developed and initially content-validated a multidimensional questionnaire assessing the clinically observable consequences of PEM in adults with ME/CFS, including functional decline, recovery dynamics, crash-associated symptoms, and pacing-related adaptations. We integrated the perspectives of individuals with lived experience of PEM and interdisciplinary experts across two iterative triangulation cycles, focusing particularly on content and face validity.

## Methods

This study used an iterative mixed-methods approach to initially validate the content of a multidimensional questionnaire designed to assess the phenotypic manifestations and systemic consequences of PEM in people with ME/CFS. The development process was informed by established guidance for PROM development, including the COSMIN methodology for content validity and methodological recommendations for scale development [14]. The qualitative components of this manuscript were reported in accordance with the Standards for Reporting Qualitative Research (SRQR), and the completed SRQR checklist is provided as supplementary material [15]. This phase primarily focused on evaluating content validity, operationalised as relevance, comprehensiveness, and comprehensibility, as well as face validity from the perspectives of people with lived experience of ME/CFS or PCS and expert stakeholders. The study protocol was approved by the ethics committee of the Medical University of Vienna (EK No. 1192/2025). All participants provided written informed consent before participation.

### Participants and recruitment

The questionnaire was evaluated by 13 adults with PEM and an interdisciplinary expert panel. Twelve of the participants had a formal physician diagnosis of ME/CFS. One participant had a formal physician diagnosis of post-COVID-19 syndrome (PCS), but not ME/CFS; however, the participant did fulfil the ME/CFS consensus criteria [16]. This participant was not recruited to represent a separate PCS subgroup. All participants were aged 18 years or older. Recruitment was conducted through an open call, in collaboration with patient organisations across the German-speaking DACH region. The aim was to achieve diversity in terms of age, sex, educational background and disease duration, rather than statistical representativeness. The degree of functional impairment was assessed using the Bell Disability Score [17]. To ensure the inclusion of individuals with severe functional impairment, flexible participation formats were offered. These included written or audio-based feedback, assistance from relatives or caregivers, and the option to split task completion over multiple shorter sessions. The expert panel included professionals from neurology, internal medicine, physiotherapy, occupational therapy, rehabilitation, and exercise science, as well as researchers with expertise in ME/CFS. The panel was selected to cover clinical and rehabilitative perspectives relevant to the assessment of PEM.

### Phase 1: item pool generation

As a first step, the authors generated an initial item pool based on a structured literature review and existing validated instruments assessing PEM, fatigue, and functional limitations. Specifically, items and domains from the DSQ-PEM [11], the CDC Symptom Inventory [18], the Chalder Fatigue Questionnaire (CFQ) [19], and FUNCAP-2712 were reviewed and adapted where appropriate. Furthermore, conceptual considerations derived from consensus documents (including the EUROMENE and D-A-CH frameworks) [16] and the International Classification of Functioning, Disability and Health (ICF) [20] were incorporated. This ensured an adequate representation of real-life functional demands and contextual factors. Based on these sources, a preliminary 13-item questionnaire was developed. The initial instrument was designed to capture four key dimensions of PEM: (1) triggers and contextual factors, (2) symptom manifestation, (3) functional limitations in daily life, and (4) temporal dynamics, including onset and recovery patterns. This version served as the foundation for subsequent evaluation.

### Phase 2: participatory evaluation with affected individuals

#### Data collection

The initial questionnaire version was evaluated through a participatory process involving individuals with lived experience of ME/CFS and PCS. Data collection employed a multimethod approach combining quantitative and qualitative components to comprehensively assess content validity, face validity, and usability from the lived experience perspective. Participants first completed a structured feedback form capturing sociodemographic characteristics, functional severity (assessed via the Bell Disability Score), and systematic evaluations of the questionnaire. Quantitative evaluation comprised eight statements (F1–F8) rated on a five-point Likert scale (1 = *strongly disagree* to 5 = *strongly agree*), assessing four quality dimensions: content relevance (F1, F2), comprehensiveness (F3, F8), comprehensibility/clarity (F4, F5), and practicability (F6, F7). Qualitative feedback was gathered through six open-ended questions addressing: (1) missing content relevant to PEM, (2) unclear or ambiguous formulations, (3) appropriateness and differentiation of response options, (4) perceived burden during completion (cognitive, emotional, physical), (5) concrete suggestions for improvement, and (6) additional comments. In addition, semi-structured interviews were conducted with four participants to explore item interpretation, cognitive response processes, and subjective experiences of PEM more in-depth. These interviews, lasting 45–60 min, were conducted via Microsoft Teams in June 2025, audio-recorded, and transcribed verbatim.

#### Data analysis

Qualitative data were analysed using structuring qualitative content analysis, following the approach outlined by Kuckartz and Rädiker [21]. A combined deductive–inductive approach was applied. Initial categories were based on the evaluation domains of content and face validity, the feedback questions, and the interview guide. These categories were expanded during analysis through inductively developed subcategories. Coding and data management were supported by MAXQDA 24 Analytics Pro (VERBI Software, Berlin, Germany). The primary coding and qualitative content analysis were conducted by TP, and TF subsequently reviewed the coding and category structure. The material was not independently double-coded. Any questions or differing interpretations identified during the review were discussed by TP and TF and resolved by consensus. Quantitative and qualitative findings were integrated through methodological triangulation after each evaluation cycle.

##### Cycle 1

In Cycle 1, questionnaire-level rating patterns were interpreted descriptively and considered alongside qualitative themes, individual comments, and feasibility considerations. As F1–F8 assessed questionnaire-level dimensions, these ratings were not used as thresholds for making decisions at item level. Decisions were guided by construct fidelity, content validity, clinical interpretability, and questionnaire economy. Additional considerations included comprehensibility, practicability, response comparability, and standardisation. The three-member core development team (AK, AAS, and TP) reviewed the integrated findings and proposed revisions during consensus meetings. When initial views differed, the relevant quantitative ratings, qualitative feedback, and rationale for the proposed revision were discussed until agreement was reached among all three members. Feedback was classified as adopted, partially adopted, not adopted, or noted, and unchanged content was retained when the integrated findings did not indicate a need for revision. All decisions and their rationales were documented in the complete revision matrices provided in Supplementary File S2. Following Cycle 1, the agreed conceptual, linguistic, and structural revisions were implemented, and the resulting questionnaire version served as the basis for the subsequent expert-panel evaluation.

### Phase 3: expert-panel content validation

The interdisciplinary expert panel subsequently evaluated the revised questionnaire. Data were collected using a structured bilingual feedback form. The quantitative evaluation comprised 16 items rated on five-point scales covering content validity, comprehensibility, practicability, methodological appropriateness, target-group specificity, and overall assessment. Experts also rated each item for relevance and clarity. Qualitative feedback was collected through open-ended response fields. Quantitative data were analysed descriptively, and qualitative comments were analysed using structuring qualitative content analysis, following Kuckartz and Rädiker [21]. Expert ratings and qualitative feedback were integrated using the same consensus-based procedure described for Cycle 1. The resulting revisions focused on conceptual consolidation, linguistic clarification, terminology standardisation, and resolution of inconsistencies. Proposed changes were adopted only when they improved construct clarity, feasibility, or standardisation without expanding the instrument beyond the core PEM construct. All Cycle 2 decisions and their rationales were documented in Supplementary File S2 (Table S2.7).

### Phase 4: linguistic review, translation and pretest

After the expert-panel revisions, the final German version of the questionnaire was reviewed by a German linguist. The questionnaire was then translated into English by a second linguist, who performed a bilingual equivalence check to ensure conceptual consistency between the German and English instrument versions. To assess the feasibility of the technical implementation, the revised questionnaire was pretested on the SoSci Survey platform with five individuals with ME/CFS attending a rehabilitation programme in Vienna (four women, one man; mean age 34 years [SD 4.9]; mean Bell Disability Score 60.0 [SD 11.5]), none of whom had participated in the Cycle 1 target-group evaluation. The aim of this phase was to identify potential issues with display, navigation and usability in an online format. Feedback from this pretest led to minor adjustments to the layout and user guidance. The resulting version constituted the revised VPEM Questionnaire used as the basis for subsequent psychometric validation.

### Provisional scoring framework

A provisional scoring framework was developed to clarify the intended interpretation of the VPEM Questionnaire and to guide subsequent psychometric evaluation. Questions 1, 2a–2e, and 5 are assigned ordered category values, with higher scores indicating greater PEM-related burden. Questions 3 and 4 are calculated as frequency-weighted mean category scores for crash severity and recovery burden, respectively. Questions 6 and 7 assess pacing use and perceived effectiveness and are interpreted separately as contextual variables. The provisional aggregate score combines Questions 1–5 and has a nominal range of 0–54. This framework represents the intended scoring structure at the conclusion of instrument development and should not be interpreted as an empirically validated scoring model; the suitability of individual components for inclusion in an aggregate score requires subsequent psychometric evaluation. The complete scoring algorithm, including worked examples, consistency requirements, and special rules for non-evaluable responses, is provided in Supplementary File S3.

### Statistical analysis

Quantitative data were analysed descriptively. Categorical variables were reported as frequencies and percentages. Continuous variables were presented as means and standard deviations or medians and interquartile ranges, depending on distribution and interpretability. Likert-scale responses from the target-group evaluation were summarised as frequencies and percentages and, for descriptive interpretation, grouped into disagreement, neutral, and agreement categories. Expert ratings were summarised across all response categories; where appropriate, the two highest response categories were combined to indicate a favourable evaluation. For item-level expert ratings, content validity was additionally quantified using the item-level Content Validity Index (I-CVI). Ratings were provided on a 5-point scale, with 1 indicating the lowest and 5 the highest rating. For relevance and clarity ratings, scores of 4 or 5 were defined a priori as favourable. An I-CVI of ≥ 0.78 was predefined as the benchmark for acceptable item-level content validity [22]. These criteria were used to identify items requiring closer review but did not automatically determine revision, retention, or exclusion; final decisions integrated the quantitative results with qualitative expert feedback. The I-CVI was calculated as the proportion of experts who provided a favourable rating for each item, with the full expert panel size (*N* = 11) as the denominator. The scale-level Content Validity Index using the average method (S-CVI/Ave) was calculated as the mean of the item-level I-CVI values across all evaluated items [22, 23].

To account for the probability of chance agreement among experts, modified kappa statistics (κ*) were calculated for each item-level relevance and clarity rating. The probability of chance agreement (Pc) was calculated as:

$$\:{P}_{c}=\frac{\mathrm{N!}}{A!\left(\mathrm{N-A}\right)!}\times\:{0.5}^{N}$$

where N represents the total number of experts and A the number of experts providing a favorable rating. Modified kappa was calculated as:

$$\:{\kappa\:}^{\mathrm{*}}=\frac{\mathrm{I-CVI}-{P}_{c}}{1-{P}_{c}}$$

Analyses were conducted using JASP 0.19.3 (JASP Team, Amsterdam, the Netherlands) and Microsoft Excel 365 (Microsoft Corporation, Redmond, WA, USA).

## Results

Table 1 shows that 13 individuals (10 female and 3 male) with a mean age of 41.9 years (SD 8.3) participated in Cycle 1. The mean Bell Disability Score was 30.0 (SD 14.1). Mean time since diagnosis was 75.5 months (SD 49.1), and mean time since symptom onset was 140.0 months (SD 93.0).

**Table 1:** Sociodemographic and clinical characteristics of the ME/CFS and PCS group (*n* = 13)

Variable	*n* (%) / Mean (SD)
Sex	
Female	10 (76.9%)
Male	3 (23.1%)
Age, years	41.9 (8.3)
Time since diagnosis, months	75.5 (49.1)
Time since symptom onset, months	140.0 (93.0)
Bell Disability Score	30.0 (14.1)
Bell Score fluctuation	18.2 (7.5)

Categorical variables are presented as frequencies and percentages (n, %). Continuous variables are presented as mean (standard deviation, SD)

### Phase 2: quantitative evaluation by individuals with PEM

**Fig. 1:**
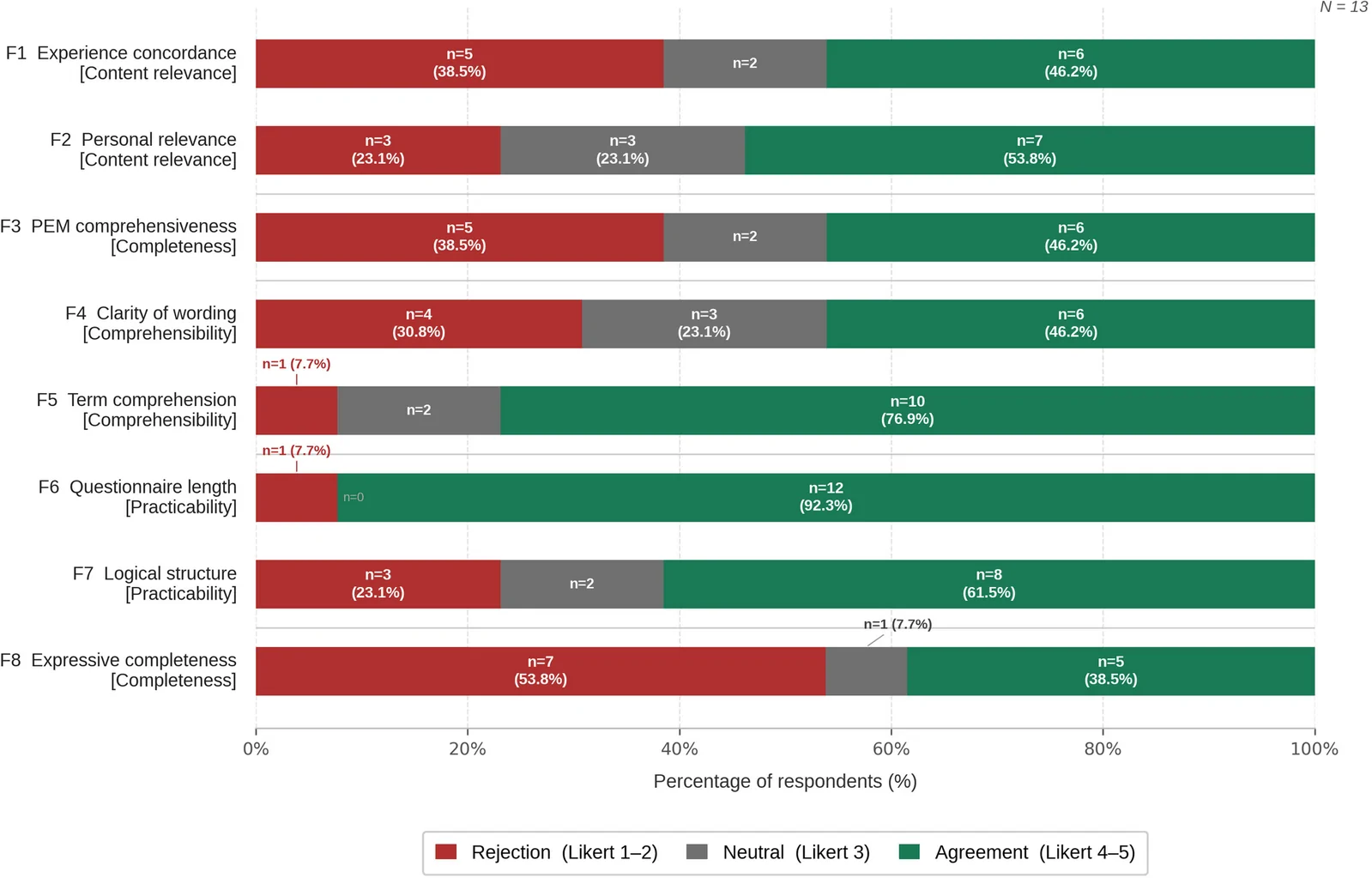
ME/CFS group assessment of the Vienna Post-Exertional Malaise Questionnaire: distribution of response categories across eight evaluation items (*N* = 13). Notes. Respondents rated each item on a 5-point Likert scale (1 = strongly disagree to 5 = strongly agree). Responses were collapsed into three categories: Rejection (Likert values 1–2), Neutral (3), and Agreement (4–5). Values within bars denote percentages; labels are omitted for segments < 10%. Abbreviations: F1 = Correspondence of the questionnaire with personal PEM experience. F2 = Relevance of the items to own ME/CFS or post-COVID syndrome experience. F3 = Comprehensiveness of PEM-related aspects covered by the questionnaire. F4 = Clarity and comprehensibility of item wording. F5 = Familiarity and comprehensibility of terminology used. F6 = Appropriateness of the overall questionnaire length. F7 = Logical and coherent ordering of items. F8 = Adequacy of response options for expressing personal PEM experiences. Quality dimensions. Content relevance: F1, F2. Completeness: F3, F8. Comprehensibility/Clarity: F4, F5. Practicability: F6, F7. Horizontal separator lines delineate dimension boundaries

Quantitative ratings across the eight evaluation items (F1–F8) from the patient cohort (*N* = 13) are presented in Fig. 1. Participants reported the highest levels of agreement on the appropriateness of the overall questionnaire length (F6: 92.3%, *n* = 12) and the comprehensibility of the terminology (F5: 76.9%, *n* = 10). Substantial agreement was also observed for the logical ordering of items (F7: 61.5%, *n* = 8) and relevance to personal experience with ME/CFS or PCS (F2: 53.8%, *n* = 7). Conversely, ratings were more heterogeneous for clarity of wording (F4: 46.2% agreement, *n* = 6; 30.8% disagreement, *n* = 4), correspondence with personal PEM experience (F1: 46.2% agreement, *n* = 6; 38.5% disagreement, *n* = 5), and coverage of relevant PEM aspects (F3: 46.2% agreement, *n* = 6; 38.5% disagreement, *n* = 5). The most critical feedback pertained to whether the questionnaire allowed for the full expression of individual PEM experiences (F8). The majority of participants disagreed with this item (53.8%, *n* = 7), while only 38.5% (*n* = 5) endorsed it.

### Qualitative findings from participants with ME/CFS or PCS

Qualitative feedback was obtained from feedback questionnaires, document annotations, semi-structured interviews, and supplementary written submissions from 13 participants with ME/CFS or PCS. Structuring content analysis yielded six main categories. Conceptual criticism was most frequent (130 segments; 11 contributors), followed by missing content (83 segments; 12 contributors), linguistic criticism (76 segments; 12 contributors), structural criticism (24 segments; 10 contributors), positive feedback (23 segments; 10 contributors), and burden of completion (9 segments; 6 contributors). Full subcategory-level results and illustrative quotations from the target population evaluation are provided in Supplementary File S1. Conceptual criticism mainly concerned the definition and representation of PEM. Participants described PEM as variable within and between individuals, including differences in crash thresholds, symptom patterns, latency, intensity, duration, and recovery. They also criticised wording that could be interpreted as equating PEM with fatigue, exhaustion, or psychological symptoms. Missing content mainly concerned trigger domains and contextual factors that participants considered insufficiently represented in the initial draft. Sensory stimuli, orthostatic load, and positively valenced emotional arousal were repeatedly identified as missing or underrepresented triggers. Additional comments referred to pacing or coping strategies, medication effects, aggravating factors and the possibility of incomplete or lasting deterioration after PEM episodes. Linguistic criticism focused on terminology and precision. Terms such as “fatigue”, “exhaustion”, “tiredness”, and “weakness” were perceived by several participants as insufficient to describe PEM. Participants also criticised vague references to exertion levels, duration or frequency and noted that some symptom descriptors did not match their experience. Structural criticism was less frequent and mainly concerned with response formats, layout, and readability. Positive feedback referred to the overall coherence, structure, and feasibility of the questionnaire. Burden of completion was reported less often and was mainly cognitive, particularly due to recalling and reflecting on recent symptom patterns.

### Phase 3: expert panel quantitative ratings

The interdisciplinary expert panel consisted of 11 professionals with substantive experience in ME/CFS clinical care and research. The medical field was represented by five clinicians, including three neurologists (27.3%) and two cardiologists (18.2%). Allied health and rehabilitation professions were represented by six experts, comprising four exercise scientists or rehabilitation therapists (36.4%), one physiotherapist (9.1%), and one occupational therapist (9.1%).

The quantitative expert ratings are presented in Tables 2 and 3. Overall, the revised questionnaire was rated favourably across all evaluated domains. For content validity, all experts rated PEM coverage as largely complete (*n* = 5, 45.5%) or complete (*n* = 6, 54.5%). The five core dimensions, PEM frequency, exertion tolerance, functional decline, recovery and new symptoms, were rated as suitable or very suitable by 10 experts (90.9%). The eight-week recall period showed more variation: four experts (36.4%) rated it as suitable or very suitable, six (54.5%) as acceptable and one (9.1%) as rather unsuitable. The comprehensibility ratings were high. Ten experts (90.9%) rated the PEM definition, examples and wording in the two highest categories, and all experts (100%) did so for the introductory texts and tables. Questionnaire length was rated as appropriate by eight experts (72.7%), the risk of triggering PEM through completion was rated as low by seven (63.6%), and response categories were rated as well differentiated by 10 (90.9%).

**Table 2:** Expert panel ratings: content validity, comprehensibility, and practicability

Dimension	Item	1 *n* (%)	2 *n* (%)	3 *n* (%)	4 *n* (%)	5 *n* (%)
Content validity	A1:Does the questionnaire cover all essential aspects of PEM? *1 = very incomplete; 5 = complete*	—	—	—	5 (45.5%)	6 (54.5%)
	A2:Are the five dimensions suitable for accurately capturing the extent and severity of PEM while avoiding false positives? *1 = very unsuitable; 5 = very suitable*	—	—	1 (9.1%)	6 (54.5%)	4 (36.4%)
	A3:Is the eight-week timeframe appropriate for capturing PEM? *1 = very unsuitable; 5 = very suitable*	—	1 (9.1%)	6 (54.5%)	1 (9.1%)	3 (27.3%)
Comprehensibility	B1:How comprehensible is the definition of PEM in item 1? *1 = very incomprehensible; 5 = very comprehensible*	—	—	1 (9.1%)	9 (81.8%)	1 (9.1%)
	B2: How comprehensible are the introductory texts and tables in items 2–6? *1 = very incomprehensible; 5 = very comprehensible*	—	—	—	10 (90.9%)	1 (9.1%)
	B3 Are the examples and wording in items 2–6 realistic and representative of the lived experience of those affected? *1 = inappropriate; 5 = very appropriate*	—	—	1 (9.1%)	6 (54.5%)	4 (36.4%)
Practicability	C1: How do you rate the questionnaire length for patients with ME/CFS? *1 = far too long; 5 = very appropriate*	—	1 (9.1%)	2 (18.2%)	8 (72.7%)	—
	C2: How high do you estimate the risk that completing the questionnaire itself could trigger PEM? *1 = very high risk; 5 = very low risk*	—	—	4 (36.4%)	7 (63.6%)	—
	C3:Are the response categories sufficiently differentiated? *1 = very inadequate; 5 = optimally differentiated*	—	—	1 (9.1%)	10 (90.9%)	—

Values are presented as *n* (%). Percentages are based on the full expert-panel sample (*N* = 11). Ratings were provided on a five-point Likert scale, with higher scores indicating greater agreement. Domains reflect content validity, comprehensibility, and practicability. Item wording was shortened in the table for readability

**Table 3:** Expert panel ratings: methodological aspects, target-group specificity, and overall appraisal

Dimension	Item	1 *n* (%)	2 *n* (%)	3 *n* (%)	4 *n* (%)	5 *n* (%)
Methodological aspects	D1:Is the non-linear score weighting appropriate for capturing PEM severity and functional limitations? *1 = not at all appropriate; 5 = very appropriate*	—	—	—	9 (81.8%)	2 (18.2%)
	D2:How do you rate the separation of crash assessment across items 3 and 4 (severity and recovery duration, respectively)? *1 = not at all meaningful; 5 = very meaningful*	—	—	—	8 (72.7%)	3 (27.3%)
	D3:Are sufficient items included to check response plausibility and identify potential inconsistencies? *1 = insufficient; 5 = very sufficient*	—	—	—	6 (54.5%)	5 (45.5%)
Target-group specificity	E1:Is the questionnaire suitable for different ME/CFS severity grades? *1 = not at all appropriate; 5 = very appropriate*	—	—	1 (9.1%)	8 (72.7%)	2 (18.2%)
	E2:Does the questionnaire adequately consider cultural and socioeconomic differences? *1 = not at all; 5 = very appropriately*	—	—	—	5 (45.5%)	6 (54.5%)
Overall appraisal	F1:How do you rate the questionnaire overall for clinical and scientific application? *1 = insufficient (fundamental reconceptualization needed); 5 = very good (ready to use)*	—	—	—	5 (45.5%)	6 (54.5%)
	F2:Would you use this questionnaire in your clinical practice or research? *1 = no*,* definitely not; 5 = yes*,* definitely*	—	—	—	5 (45.5%)	6 (54.5%)

Values are presented as *n* (%). Percentages are based on the full expert-panel sample (*N* = 11). Ratings were provided on a five-point Likert scale, with higher scores indicating greater agreement. Domains reflect methodological appropriateness, target-group specificity, and overall appraisal. Item wording was shortened in the table for readability

Methodological aspects received uniformly positive ratings. All experts rated the proposed score weighting, the separation of crash severity and recovery duration, and the plausibility checks in the two highest response categories. Target-group specificity was similarly supported: 10 experts (90.9%) rated the questionnaire as suitable or very suitable for different ME/CFS severity levels, and all experts rated its consideration of cultural and socioeconomic differences as appropriate or very appropriate. Overall appraisal was high. All experts rated the questionnaire as good or very good for clinical and scientific use; six (54.5%) supported use in practice or research without reservation and five (45.5%) would use it after minor adaptations.

The results of the content validation at the item level (I-CVI) and scale level (S-CVI/Ave), as well as the modified kappa values (k*) for relevance and clarity, are presented in Tables 4 and 5. All items met the predefined I-CVI benchmark of ≥ 0.78 for the relevance dimension. Questions 1 (Post-exertional deterioration), 2 (Exertion tolerance), 3 (Number of crashes), and 6 (Pacing) were rated as relevant or highly relevant by all 11 experts (I-CVI = 1.00). Question 4 (Recovery capacity) received a favourable rating from 10 experts (I-CVI = 0.91), and Question 5 (New symptoms) from nine experts (I-CVI = 0.82). The S-CVI/Ave for relevance was 0.96, indicating excellent validity at the instrument level. The modified kappa values confirmed these results (k* ranging from 0.81 to 1.00), pointing to excellent agreement beyond chance. Clarity ratings showed greater variability (I-CVI range: 0.55–0.91). While Questions 1, 3, and 6 achieved high scores (I-CVI = 0.91), the scores for Questions 2 and 4 were slightly lower but acceptable at 0.82 each. Question 5 (New symptoms) fell below the predefined threshold, receiving only six favourable ratings (I-CVI = 0.55; k* = 0.41). This indicates a need for linguistic and structural refinement of this item. The S-CVI/Ave for clarity was 0.82.

**Table 4:** Item-level ratings for relevance and clarity by the expert panel (*N* = 11)

Item	Dimension	Score 5 *n* (%)	Score 4 *n* (%)	Score 3 *n* (%)	Score 2 *n* (%)	Score 1 *n* (%)
Question 1: Post-exertional deterioration	Relevance	7 (63.6)	4 (36.4)	0 (0.0)	0 (0.0)	0 (0.0)
	Clarity	4 (36.4)	6 (54.5)	1 (9.1)	0 (0.0)	0 (0.0)
Question 2: Exertion tolerance	Relevance	8 (72.7)	3 (27.3)	0 (0.0)	0 (0.0)	0 (0.0)
	Clarity	3 (27.3)	6 (54.5)	2 (18.2)	0 (0.0)	0 (0.0)
Question 3: Number of crashes	Relevance	7 (63.6)	4 (36.4)	0 (0.0)	0 (0.0)	0 (0.0)
	Clarity	3 (27.3)	7 (63.6)	1 (9.1)	0 (0.0)	0 (0.0)
Question 4: Recovery capacity	Relevance	6 (54.5)	4 (36.4)	1 (9.1)	0 (0.0)	0 (0.0)
	Clarity	5 (45.5)	4 (36.4)	1 (9.1)	1 (9.1)	0 (0.0)
Question 5: New symptoms	Relevance	6 (54.5)	3 (27.3)	2 (18.2)	0 (0.0)	0 (0.0)
	Clarity	4 (36.4)	2 (18.2)	5 (45.5)	0 (0.0)	0 (0.0)
Question 6: Pacing	Relevance	11 (100.0)	0 (0.0)	0 (0.0)	0 (0.0)	0 (0.0)
	Clarity	9 (81.8)	1 (9.1)	1 (9.1)	0 (0.0)	0 (0.0)

**Table 5:** Item-level Content Validity Index and modified kappa for relevance and clarity (*N* = 11)

Item	Dimension	Favourable ratings *n*/11	I-CVI	Pc	κ*
Question 1: Post-exertional deterioration	Relevance	11/11	1.00	0.0005	1.00
	Clarity	10/11	0.91	0.0054	0.91
Question 2: Exertion tolerance	Relevance	11/11	1.00	0.0005	1.00
	Clarity	9/11	0.82	0.0269	0.81
Question 3: Number of crashes	Relevance	11/11	1.00	0.0005	1.00
	Clarity	10/11	0.91	0.0054	0.91
Question 4: Recovery capacity	Relevance	10/11	0.91	0.0054	0.91
	Clarity	9/11	0.82	0.0269	0.81
Question 5: New symptoms	Relevance	9/11	0.82	0.0269	0.81
	Clarity	6/11	0.55	0.2256	0.41
Question 6: Pacing	Relevance	11/11	1.00	0.0005	1.00
	Clarity	10/11	0.91	0.0054	0.91
S-CVI/Ave	**Relevance**	**—**	**0.96**	**—**	**—**
S-CVI/Ave	**Clarity**	**—**	**0.82**	**—**	**—**

Favourable ratings were defined as scores of 4 or 5. The item-level Content Validity Index (I-CVI) was calculated as the proportion of experts providing a favourable rating for each item. The probability of chance agreement (Pc) was calculated using the binomial probability formula. Modified kappa (κ*) was calculated to adjust the I-CVI for chance agreement. The scale-level Content Validity Index using the average method (S-CVI/Ave) was calculated as the mean of the item-level I-CVI values across all evaluated items

*Abbreviations*: *I-CVI* item-level Content Validity Index, *Pc* probability of chance agreement, *S-CVI/Ave* scale-level Content Validity Index using the average method, κ* modified kappa

### Iterative instrument refinement

The questionnaire was revised in two cycles. In both cycles, quantitative ratings and qualitative feedback were considered jointly and discussed by the three-member core development team. For each major feedback point, the team decided whether it should be adopted, partially adopted, documented, or not implemented. The complete revision matrices for triangulation cycles 1 and 2 are provided in Supplementary File S2.

#### Cycle 1: revisions following feedback from individuals with ME/CFS or PCS

Feedback from participants with lived experience led to a substantial revision of the initial 13-item draft. Ambivalent ratings of content fit and completeness were explained by qualitative feedback indicating that the initial draft was perceived as overly aligned with general fatigue, exhaustion, or psychological symptoms. PEM was therefore described throughout the questionnaire not as a collection of individual symptoms, but as a disproportionate deterioration after exertion. Psychological symptoms were removed from the core construct, potentially stigmatising terms were deleted, and wording was aligned more closely with participants’ lived experience. Missing trigger domains were also added. Sensory or environmental stimuli, orthostatic load, and positively valenced emotional triggers were incorporated into the exertion matrix. Pacing was added as a contextual item because low crash frequency may also reflect strict activity limitation. A simple yes/no PEM screening item was not adopted to avoid false-negative self-classification among individuals with uncertain symptom attribution. Feedback on medication effects, comorbidities, social security, and PEM in children or adolescents was documented but not included in the core questionnaire because these aspects extend beyond the PEM construct. Instrument structure was also revised. The questionnaire was reduced from 13 items to five core questions, response options were clarified, and the layout was simplified. Structured count-based formats were introduced to capture different crash patterns, allowing crash severity and recovery duration to be assessed in a differentiated but standardised way. After the first revision, the questionnaire assessed five dimensions: frequency, exertion tolerance, functional decline, recovery capacity, and new symptoms, supplemented by a non-scored pacing item.

#### Cycle 2: revisions following expert-panel evaluation

After the first revision, the expert panel supported the overall structure of the instrument, particularly the multidimensional assessment of PEM, the proposed score weighting, and the separate assessment of crash severity and recovery duration. Thus, it was not required to conduct a fundamental redesign. The second revision focused on targeted refinements, terminology standardisation and resolution of specific inconsistencies. Delayed onset was no longer formulated as a mandatory criterion, but as a frequent feature of PEM. At the same time, the disproportionate response to exertion was emphasised. Social exertion was integrated into the emotional-social trigger category. The term “crash” was used consistently, and technical or potentially ambiguous wording was replaced or supplemented with plain-language formulations. An inconsistency between the definition’s fixed minimum duration of PEM and the shorter recovery-duration response options was resolved by removing the fixed minimum-duration criterion. A free-text response option was not implemented because it could have reduced the level of standardisation and increased completion burden. Also, a two-part version for different severity levels was not developed, as this would have required a separate development and validation phase. After the second revision, the instrument was designated the VPEM Questionnaire. The final version retained the five core PEM dimensions and added two non-scored pacing context items assessing pacing use and perceived effectiveness.

### Phase 4: pretest results

The pre-test was completed by five participants on the online SoSci Survey platform. No major comprehension problems were identified. Two technical and presentational issues emerged. First, the online layout of the exertion matrix in item 2 was rated as difficult to navigate in its original single-page format; the matrix was therefore distributed across five separate pages, one per trigger domain, to improve readability and reduce the burden of scrolling. Second, participants indicated that it was unclear which fields in item 3 required completion; an instruction was added specifying that only the fields corresponding to experienced crash intensities should be filled in, with all remaining fields left blank. Following these adjustments, the instrument was finalised as the VPEM Questionnaire. The final English and German questionnaire versions and the provisional scoring framework are provided in Supplementary File S3.

## Discussion

This study used an iterative, exploratory sequential mixed-methods approach to conduct the initial content validation of the VPEM Questionnaire, a multidimensional questionnaire designed to assess the clinically observable consequences of PEM in individuals with ME/CFS or PCS with PEM. The instrument captures five core PEM dimensions - crash frequency, exertion thresholds across five trigger classes, functional impact, recovery dynamics, and new crash-associated symptoms - supplemented by non-scored pacing context items. Patient and expert-panel data converged on the need to move beyond frequency- and severity-centred approaches to PEM assessment. The evaluation by the ME/CFS and PCS cohort identified conceptual ambiguities in the initial draft, particularly regarding the distinction between PEM, nonspecific fatigue and psychological symptoms, as well as insufficient coverage of trigger diversity and intra-individual variability. Expert ratings of the revised version were generally favourable, particularly regarding content coverage, intelligibility, methodological design, and overall suitability, thereby supporting the instrument’s content and face validity at this stage of development. A key finding from the patient evaluation was the identification of conceptual ambiguities in the primarily literature-based initial draft. In particular, insufficient differentiation from nonspecific fatigue and psychological symptoms, missing trigger domains, and inadequate representation of intra-individual variability indicated that the patient perspective is not merely complementary but methodologically central to the development of a content-valid PROM [24].

### Multidimensionality of PEM

Our findings highlight that PEM cannot be adequately represented along a single dimension. Differentiated assessment of trigger types, functional impact, recovery dynamics and associated new symptoms is required to reflect the clinical reality of individual patients. This aligns with recent work describing PEM as a multi-layered phenomenon characterised by diverse trigger categories, frequently delayed onset, variable recovery trajectories, multisystem symptomatology, marked functional impairment and prolonged or incomplete recovery [25–27]. A purely symptom-focused assessment, therefore, risks missing core characteristics of PEM [9]. At the same time, this broader conceptual coverage exposes a tension between content breadth and feasibility. ME/CFS participants described experiencing cognitive, emotional and occasionally physical strain when completing the instrument, with retrospective recall proving particularly challenging. As completing a PEM instrument may exacerbate symptoms in some individuals, the development of such instruments must balance conceptual differentiation with practical tolerability.

### Conceptual positioning of PEM

Although PEM is often described in clinical criteria as a cardinal symptom of ME/CFS [9], the feedback obtained in this study suggested that a multidimensional questionnaire approach would be more appropriate than relying on a single symptom item. Rather, PEM represents a dynamic post-exertional deterioration pattern characterised by disproportionate exacerbation of symptoms, functional decline, delayed onset in many cases and impaired or incomplete recovery [27, 28]. Therefore, its clinical meaning lies not only in the occurrence of symptoms after exertion, but also in the interaction among exertional triggers, multisystemic deterioration, loss of function, and the inability to return to the pre-exertional baseline promptly [5, 28]. In terms of instrument development, this implies that PEM should be assessed as a multidimensional illness process rather than a unidimensional symptom [5].

The VPEM Questionnaire was developed to assess the clinically observable consequences of post-exertional deterioration rather than PEM as a single symptom item. It covers several interrelated aspects of this deterioration, including crash frequency, exertion thresholds across trigger domains, functional impact, recovery dynamics, new crash-associated symptoms, and pacing context.

### Construct demarcation: fatigue and psychological symptoms

Inadequate construct demarcation emerged as a central methodological issue for content validity in the first cycle. This was particularly evident for the term “fatigue”. Target-group feedback and existing literature indicate that fatigue is a non-specific symptom occurring across many chronic conditions and does not adequately capture the specific phenomenon of exertion intolerance in ME/CFS [7, 29]. The distinction between PEM and psychological symptoms also proved highly relevant. Wording that places PEM close to affective instability, burnout or primarily psychological exhaustion may contribute to misclassification and reinforce existing experiences of stigma [30]. The VPEM Questionnaire addresses this risk through patient-near language, particularly the use of the term “crash”, everyday examples, and the avoidance of psychologising formulations. These linguistic choices are methodological rather than merely stylistic: many terms may be formally understood but still fail to reflect the lived experience of PEM. In this context, linguistic precision is a prerequisite for content validity, acceptability, and valid application of the instrument [31, 32]. Patient-near, non-stigmatising language may also be clinically relevant, as people with ME/CFS frequently report lack of recognition of their symptoms, with implications for trust and the therapeutic relationship [30, 33].

These findings also require a critical appraisal of initial screening items used in some shorter instruments. A single filter question that terminates the questionnaire when answered negatively assumes that respondents can already identify their experience as PEM. This may be problematic, particularly for newly affected or diagnostically uncertain individuals, and may increase the risk of false-negative self-classification [11].

### Trigger domains and exertion thresholds

In developing the VPEM Questionnaire, we captured how post-exertional deterioration manifests across different types of everyday exertion. We therefore did not conceptualise PEM as a single symptom item or rely solely on a global severity rating. Instead, we aimed to operationalise PEM through the response to exertion: which types of exertion typically trigger a crash, at what exertion threshold this occurs, how strongly daily functioning is impaired, and whether additional symptoms emerge during a crash. This approach was based on the understanding that PEM is clinically characterised by an abnormal and disproportionate response to exertion, often involving delayed symptom exacerbation and prolonged recovery [13]. The same deterioration pattern may become visible after different forms of exertion, including physical, cognitive, sensory, environmental, emotional-social, and orthostatic demands [9, 11]. Therefore, we categorised these trigger domains and provided everyday examples to help respondents identify the level of exertion at which a crash is likely to occur. Accordingly, crash severity and recovery were assessed across crashes rather than separately by trigger domain, because individual crashes may not always be reliably attributed to a single trigger. Nevertheless, the questionnaire was designed to capture the typical exertion thresholds at which specific types of everyday activity are likely to contribute to post-exertional deterioration. The examples were arranged as graded functional anchors, drawing on the ICF framework and function-related instruments reviewed during item development. Basic self-care and brief everyday activities represented lower functional demands than prolonged mobility, sustained cognitive activity, or more complex social and sensory exposure [34]. For the non-physical domains, the gradation reflected increasing duration, intensity, and complexity and was refined through feedback from individuals with lived experience and the expert panel. The examples were intended to anchor responses to the functional demands of an activity rather than to subjective effort alone and were explicitly presented as orientation aids rather than fixed or universally equivalent thresholds. Orthostatic load was treated as a distinct domain because target-group feedback and prior evidence suggest that upright posture or positional change may trigger post-exertional deterioration independently of physical activity [35].

Crashes often result from cumulative rather than single exposures.We assessed pacing separately because it can modify the observable expression of PEM. A low crash frequency may indicate milder disease, but it may also reflect strict activity restriction and successful avoidance of exertion thresholds [11]. Against this background, the eight-week recall window was chosen as a pragmatic compromise between capturing recurrent or episodic PEM and reducing retrospective burden. A shorter period might result in crash susceptibility being underrepresented in individuals who temporarily remain below their exertion threshold [36], whereas, a longer period may increase detailed recall burden. General guidance indicates that no single recall period is optimal and that its selection should reflect the frequency, variability, and duration of the phenomenon assessed [37]. Although a six-month period showed favourable reliability for global ME/CFS symptom ratings, including PEM, this does not establish the reliability of recalling individual crashes by frequency, severity, and recovery duration [38]. The eight-week recall period should therefore be regarded as provisional at this stage of questionnaire development. Its suitability will be examined in the subsequent larger psychometric validation, and a shorter recall window may be adopted if it improves response quality and interpretability without compromising the capture of clinically relevant PEM patterns. Digital symptom diaries or high-frequency monitoring may complement retrospective instruments by capturing short-term fluctuations and individual exertion–symptom relationships in greater detail [39].

### Positioning in relation to existing instruments

The VPEM Questionnaire is complementary to, and does not replace existing instruments. Existing PEM instruments, such as the DSQ-PEM and DSQ-PEM-2, provide established approaches to assessing PEM-related symptoms and selected PEM characteristics, including frequency, severity, duration, triggers and pacing-related aspects [7, 11]. Its distinction lies in its focus on the clinically observable consequences of post-exertional deterioration rather than on fatigue severity or functional capacity alone. Specifically, it links exertion thresholds across trigger domains with crash occurrence, functional decline, recovery dynamics, new crash-associated symptoms, and pacing context within a single assessment framework.

### Limitations

Several limitations should be considered. First, the initial item pool was primarily developed based on existing literature, consensus documents, and established instruments. Individuals with lived experience of the disease were systematically involved in the evaluation and revision of the first draft, but not in a formal co-generation phase before the creation of the initial item pool. Earlier patient involvement might have expanded the initial item pool and pre-empted some of the conceptual revisions required later. Second, the target-group sample was small and predominantly recruited via patient organisations. While this approach was suitable for obtaining detailed feedback from individuals highly experienced with PEM, it may have introduced a selection bias towards individuals with high health literacy, profound symptom reflection, and strong motivation to participate in questionnaire development. Even though there were flexible participation formats, the perspectives of individuals who are newly ill, patients with lower health literacy or from different socioeconomic backgrounds, and those with milder or very severe disease may not have been fully captured. Third, the expert panel did not include all initially targeted professional perspectives. Specifically, experts in psychometrics, questionnaire development, and nursing science were absent. Consequently, psychometric design issues, nursing perspectives, and questions regarding implementation in various care contexts could only be considered to a limited extent. Fourth, the qualitative coding and revision process necessarily involved interpretative decisions. TP conducted the primary coding, while TF reviewed the coding and category structure; however, the material was not independently double-coded. Although differing interpretations were discussed and all major revision decisions were documented in revision matrices, the absence of independent duplicate coding may have increased the influence of individual interpretation on the findings and final instrument structure. Fifth, the quantitative ratings, including I-CVI and modified kappa values, should be interpreted as descriptive indicators of expert agreement rather than formal psychometric validation. While these metrics are valuable for content validation, they depend on the panel’s composition and size, as well as the chosen rating thresholds. In addition, although the exertion examples were designed as graded functional anchors, respondents may perceive the demand of individual activities differently. Therefore, some residual variation in how these examples are interpreted cannot be excluded and should be examined further during psychometric validation. Finally, different versions of the instrument were evaluated in Cycles 1 and 2, precluding direct numerical comparison between the cycles. The post-revision version was not subjected to a repeat expert-panel content-validity assessment; therefore, improvements in I-CVI and S-CVI/Ave could not be confirmed. Several wording and example refinements introduced during peer review were likewise not part of the original expert-panel or pretest assessment and will require re-evaluation in the next validation round.

### Future research

Further work should psychometrically evaluate the revised VPEM Questionnaire in a larger cohort of individuals with ME/CFS, including reliability, construct validity, score interpretation, and comparison with established instruments such as the DSQ-PEM and FUNCAP-27. Particular attention should be given to the optimal recall period and to the interpretation of the graded exertion anchors. Question 5 will also be refined and cognitively tested to distinguish newly occurring symptoms that resolve with the crash from those that persist beyond it. Further work should examine discriminant validity in other conditions characterised by fatigue, such as multiple sclerosis, to assess the ability of the instrument to distinguish PEM-related post-exertional deterioration from non-specific fatigue.

## Conclusion

A systematic, iterative, and multi-perspective development process resulted in the VPEM Questionnaire, a multidimensional instrument assessing PEM across crash frequency, exertion thresholds in five trigger domains, functional impairment, recovery dynamics, new crash-associated symptoms, and non-scored pacing context items. Feedback from patients and experts supports the initial content and face validity of the evaluated VPEM development versions and provides a conceptually grounded basis for further refinement and psychometric validation.

## Supplementary Information

Supplementary Material 1.

Supplementary Material 2.

Supplementary Material 3.

Supplementary Material 4.

Supplementary Material 5.

## Data Availability

The anonymised feedback data are available from the corresponding author upon reasonable request.

## Declarations

### Patient, public, or community engagement

Individuals with lived experience of ME/CFS were involved in the evaluation and iterative refinement of the VPEM Questionnaire. They contributed through structured ratings, open-ended written feedback, document annotations, and semi-structured interviews. Their input directly informed conceptual, linguistic, and structural revisions of the instrument, including construct demarcation, patient-near terminology, trigger domains, response formats, and pacing-related context. Participants were not involved as co-investigators in the initial item generation, data analysis, or dissemination planning.
