# Supplementary material for: Development and initial content validation of the Vienna post-exertional malaise questionnaire: an iterative mixed-methods study
Source: Qual Life Res. 2026 Nov 7;35(12):327. doi: 10.1007/s11136-026-04434-2 (PMC13744550; doi:10.1007/s11136-026-04434-2)
Supplement: Supplementary file 1 [file 11136_2026_4434_MOESM1_ESM.pdf]

The SRQR reporting checklist

Completed for: Development and initial content validation of the Vienna Post-Exertional Malaise Questionnaire: an iterative mixed-methods study

Note. This checklist follows the SRQR reporting checklist structure shown in the provided PDF. Locations refer to the current VPEM manuscript version and should be checked again after journal-specific formatting and any revision of the manuscript.

	Item Description	Location (or reason for not reporting)
**Title & Abstract**		
Title	Describe the nature and topic of the study. Identify the study as qualitative or indicate the approach or data collection methods.	Title page (p. 1): identifies the study as an iterative mixed-methods study and names the VPEM Questionnaire.
Abstract	Summarise the key elements of the study using the abstract format of the intended publication.	Abstract (pp. 2-3): objectives, methods, results, and conclusions.
**Introduction**		
Problem Formulation	Describe the problem/phenomenon studied, its significance, relevant theory and empirical work, and gaps in current knowledge.	Introduction (pp. 4-5): PEM in ME/CFS and Long COVID, limitations of existing PEM instruments, and need for a multidimensional patient-reported measure.
Purpose or research question	Describe the purpose of the study and specific objectives or questions.	Introduction, final paragraph (p. 5): development and initial content validation of a multidimensional questionnaire assessing clinically observable consequences of PEM.
**Methods**		
Qualitative approach and research paradigm	Describe your qualitative approach, your guiding theory (if appropriate), and research paradigm, and reasons for your choices.	Methods (pp. 5-8): iterative mixed-methods instrument-development design informed by COSMIN guidance; qualitative component analysed using structuring qualitative content analysis with a deductive-inductive approach.
Researcher characteristics and reflexivity	Describe how researchers' characteristics may influence the research, including personal attributes, qualifications/experience, relationship with participants, assumptions, and/or presuppositions; potential or actual interaction between researchers' characteristics and the research questions, approach, methods, results and/or transferability.	Partly reported. Methods describe the interdisciplinary development process and consensus meetings (pp. 7-8); author contributions describe roles of AK, AAS, TP and TF (p. 19). A formal reflexivity statement is not reported explicitly in the current manuscript.
Context	Describe the setting/site(s) in which the study was conducted, why it was selected, and any other salient contextual factors that may influence the study.	Methods; Participants and recruitment (pp. 5-6): Medical University of Vienna ethics context, recruitment through patient organisations, flexible participation formats, and interdisciplinary expert panel.
Sampling strategy	Describe how and why research participants, documents, or events were selected; criteria for deciding when no further sampling was necessary, and the rationale for those criteria.	Methods; Participants and recruitment (pp. 5-6): adults with ME/CFS or Long COVID with PEM; recruitment via patient organisations to maximise heterogeneity; expert panel selected to cover clinical and rehabilitative perspectives.
Ethical issues pertaining to human subjects	Describe any approval by an appropriate ethics review board and participant consent, or explain any lack thereof. Describe any other confidentiality and data security issues.	Methods (p. 5) and Declarations/Ethics approval (p. 18): Ethics Committee of the Medical University of Vienna, EK No. 1192/2025; written informed consent obtained from all participants. Data availability and anonymised feedback data described in Declarations (p. 18).
Data collection methods	Describe the types of data collected; details of data collection procedures including (as appropriate) start and stop dates of data collection and analysis, iterative process, triangulation of sources/methods, and modification of procedures in response to evolving study findings. Describe your rationale for these choices.	Methods; Phase 1-4 (pp. 6-8): item generation, structured feedback forms, Likert ratings, open-ended feedback, document annotations, semi-structured interviews in June 2025, expert-panel evaluation, linguistic review, translation, and online pretest. Iterative revisions and triangulation are described in Methods and Results (pp. 7-13).
Data collection instruments and technologies	Describe any instruments (e.g., interview guides, questionnaires) and devices (e.g., audio recorders) used for data collection; describe if/how the instrument(s) changed over the course of the study.	Methods; Phase 1-4 (pp. 6-8): initial 13-item questionnaire draft, structured feedback form, Likert scales, open-ended questions, semi-structured interview guide, Microsoft Teams audio-recording and verbatim transcription, expert feedback form, SoSci Survey pretest. Revisions from 13-item draft to final VPEM Questionnaire described in Results (pp. 11-13).
Units of study	Describe the number and relevant characteristics of participants, documents, or events included in the study. Describe the level of participation.	Results and Table 1 (pp. 9, 20): 13 individuals with ME/CFS or Long COVID with PEM; expert panel characteristics (p. 10); pretest with five participants (p. 13).

	Item Description	Location (or reason for not reporting)
Data processing	Describe the methods for processing data prior to and during analysis, including transcription, data entry, data management and security, verification of data integrity, data coding, and anonymisation / deidentification of excerpts.	Methods; Data analysis and Statistical analysis (pp. 7-9): interviews audio-recorded and transcribed verbatim; coding and data management in MAXQDA 24; revision matrices; quantitative analyses in JASP and Microsoft Excel. Confidentiality/anonymised data described in Declarations (p. 18). Data security procedures are not described in detail.
Data analysis	Describe the process by which inferences, themes, etc. were identified and developed, including the researchers involved in data analysis; usually references a specific paradigm or approach. Describe why you chose this process.	Methods; Data analysis (p. 7): structuring qualitative content analysis following Kuckartz and Raediker using a combined deductive-inductive approach; integration of quantitative and qualitative findings through methodological triangulation; consensus decisions by a three-member core development team. Statistical analyses described on pp. 8-9.
Techniques to enhance trustworthiness	Describe any techniques to enhance trustworthiness and credibility of data analysis,(e.g., member checking, triangulation, audit trail). Describe why you chose these techniques.	Methods (pp. 7-8) and Results (pp. 11-13): triangulation of quantitative ratings, qualitative themes, comments and feasibility considerations; consensus meetings; revision matrices documenting adopted, partially adopted, not adopted or noted feedback; expert-panel review; pretest. No formal member checking is reported.
**Results**		
Synthesis and interpretation	Describe the main findings (e.g., interpretations, inferences, and themes); might include development of a theory or model, or integration with prior research or theory.	Results (pp. 9-13): quantitative patient ratings, six qualitative feedback categories, expert-panel ratings, item-level content validity indices, modified kappa values, and iterative instrument refinement.
Links to empirical data	Provide evidence (e.g., quotes, field notes, text excerpts, photographs) to substantiate analytic findings.	Results (pp. 9-13), Figure 1 (p. 21), Tables 1-5 (pp. 20-25), and Supplementary File S1 containing qualitative coding summaries and illustrative quotations.
**Discussion**		
Integration with prior work, implications, transferability, and contribution(s) to the field	Summarize the main findings, explain how findings and conclusions connect to, support, elaborate on, or challenge conclusions of earlier scholarship; discuss the scope of application/generalizability; identify unique contribution(s) to scholarship in a discipline or field.	Discussion (pp. 13-17): multidimensionality of PEM, construct demarcation from fatigue and psychological symptoms, intra-individual variability, recall window, positioning relative to DSQ-PEM/DSQ-PEM-2 and FUNCAP, and implications for future psychometric validation. Conclusion (pp. 17-18).
Limitations	Discuss the trustworthiness and limitations of findings	Discussion; Limitations (pp. 16-17): initial item-pool generation, small and patient-organisation recruited sample, missing professional perspectives, interpretative decisions by the core team, descriptive nature of I-CVI/kappa values, and different instrument versions across cycles.
**Other**		
Conflicts of interest	Describe any potential sources of influence or perceived influence on study conduct and conclusions. Describe how these were managed.	Declarations; Competing interests (p. 18): authors declare no competing interests.
Funding	Describe sources of funding and other support. Describe the role of funders in data collection, interpretation, and reporting.	Declarations; Funding (p. 18): no external project funding for the present validation study; compensation payments for the preceding questionnaire-development phase supported by ZonMw Onderzoeksprogramma ME/CVS and post-COVID. No funder role in data collection, interpretation or reporting is stated.

Citation recommended by SRQR: O'Brien BC, Harris IB, Beckman TJ, Reed DA, Cook DA. Standards for reporting qualitative research: A synthesis of recommendations. Academic Medicine. 2014;89(9):1245-1251.
The provided SRQR checklist PDF also recommends including the completed checklist as a supplement and describing use of SRQR at the end of the Methods section.
