# Supplementary material for: Development and initial content validation of the Vienna post-exertional malaise questionnaire: an iterative mixed-methods study
Source: Qual Life Res. 2026 Nov 7;35(12):327. doi: 10.1007/s11136-026-04434-2 (PMC13744550; doi:10.1007/s11136-026-04434-2)
Supplement: Supplementary file 2 [file 11136_2026_4434_MOESM2_ESM.docx]

**Supplementary File S1**

**Phase 2: Quantitative evaluation by individuals with ME/CFS and PCS**

This supplementary file provides the complete subcategory-level results of the qualitative content analysis conducted during the target-group evaluation phase (Phase 1). Data were obtained from N = 13 participants (P01–P13) and one additional anonymous contributor, across feedback questionnaires, PDF annotations, semi-structured interviews, and supplementary written submissions. Results are organised by main category, with subcategory frequency tables and illustrative participant quotations. All quotations were translated from German; original-language versions are available from the corresponding author on request.

**S1.1 Overview of Main Categories**

Table S1.1 presents the six main categories identified in the qualitative content analysis, with the number of coded segments and contributing participants per category.

| **Main category** | **Segments (n)** | **Participants (n)** |
| --- | --- | --- |
| **Conceptual criticism** | 130 | 11 |
| **Missing content** | 83 | 12 |
| **Linguistic criticism** | 76 | 12 |
| **Structural criticism** | 24 | 10 |
| **Positive feedback** | 23 | 10 |
| **Burden of completion** | 9 | 6 |

*Note. One segment denotes one coded text unit; a participant may have contributed multiple segments within or across categories.*

**S1.2 Conceptual Criticism**

Conceptual criticism was the most frequently coded main category (130 segments; 11 participants). Subcategory details are presented in Table S1.2, followed by representative participant quotations for each subtheme.

| **Subcategory** | **Segments (n)** | **Participants (n)** |
| --- | --- | --- |
| **Conceptual criticism (total)** | 130 | 11 |
| Heterogeneity / variability of PEM | 28 | 11 |
| Definitional boundary: PEM vs fatigue/exhaustion | 27 | 8 |
| Definitional boundary: PEM vs psychological symptoms | 8 | 5 |
| Definitional boundary: PEM as mechanism, not symptom | 6 | 3 |
| Temporal characteristics (latency / duration) | 17 | 6 |
| Inadequate representation of symptom pattern | 16 | 8 |
| Risk of stigmatisation | 15 | 5 |
| Unclear rating scale (scoring) | 8 | 6 |
| Redundancy / content overlap | 5 | 2 |

*Note. Segments could be assigned to multiple subcategories; subcategory totals therefore exceed the main category total.*

**Heterogeneity and variability of PEM**

Participants described crash profiles as highly variable both within and across individuals, with differences in onset latency, intensity, symptom constellation, and recovery duration. Several respondents emphasised that the instrument must be sensitive to both mildly and severely affected individuals.

*“What should mildly affected individuals answer — those who do experience PEM, but only after more substantial exertion — and, on the other hand, severely and most severely affected individuals who are no longer able to perform these activities at all?”* — P12, supplementary document

*“Since this is about PEM, a comparison between a ‘good day’ and a ‘bad day’ would be of particular interest.”* — Additional anonymous contributor, PDF annotations

**Definitional boundaries: PEM vs fatigue and exhaustion**

Eight participants argued that PEM must be clearly distinguished from non-specific fatigue or exhaustion, noting that items framing PEM primarily through the concept of exhaustion were conceptually inaccurate.

*“It is generally noticeable that PEM is always defined first through exhaustion and an increase in exhaustion — but that is not correct.”* — P07, feedback questionnaire

**Definitional boundaries: PEM vs psychological symptoms**

Five participants raised concerns that certain items implied mood disturbance as a characteristic feature of PEM. Participants considered this inaccurate and potentially stigmatising, as emotional symptoms were perceived as secondary rather than cardinal manifestations of ME/CFS.

*“Low mood has never been something I have heard of as a primary symptom of ME. Of course, as with any other severely disabling condition, depression can occur secondarily — but it is secondary, and therefore not a primary, characteristic ME symptom.”* — P13, feedback questionnaire

*“The mention of low mood reproduces stigmas […] with the risk of re-traumatising those affected.”* — P12, supplementary document

**Temporal characteristics**

Six participants highlighted that the delayed onset of symptom exacerbation — a key diagnostic feature — was insufficiently captured. Concerns included the absence of items on latency between exertion and symptom onset, the duration of the worsened symptom phase, and systematic crash frequency. One participant noted that symptom worsening occurring more than 14 hours after exertion should be required for a PEM classification.

**Inadequate representation of symptom pattern**

Eight participants noted ambiguity regarding whether items referred to baseline symptomatology or to PEM-triggered exacerbations, a distinction considered essential for avoiding inconsistent responses.

**Risk of stigmatisation**

Five participants expressed concern that certain terms — including Erschöpfung (exhaustion), Müdigkeit (fatigue), and Burnout — carried stigmatising connotations and could contribute to misattribution of ME/CFS symptomatology.

**Unclear scoring and redundancy**

Six participants described the scoring categories at the end of the questionnaire (e.g., ‘mild’, ‘moderate’) as confusing or misleading. Two participants identified content overlap between individual items.

**S1.3 Missing Content**

Twelve participants contributed 83 coded segments identifying content absent from or insufficiently represented in the initial item set (Table S1.3).

| **Subcategory** | **Segments (n)** | **Participants (n)** |
| --- | --- | --- |
| **Missing content (total)** | 83 | 12 |
| PEM triggers: sensory stimuli | 17 | 8 |
| PEM triggers: orthostatic load | 12 | 5 |
| PEM triggers: positive emotional arousal | 9 | 5 |
| Symptoms (additional) | 16 | 7 |
| Screening item (PEM yes/no) | 6 | 3 |
| Coping strategies / pacing | 5 | 3 |
| Medication effects on PEM | 5 | 2 |
| Exacerbating / associated factors | 5 | 5 |
| Irreversibility of PEM | 4 | 4 |
| Comorbidities | 2 | 2 |
| Social security / support | 1 | 1 |
| PEM in children and adolescents | 1 | 1 |

*Note. Segments could be assigned to multiple subcategories.*

**PEM triggers: sensory, orthostatic, and emotional**

The most consistently identified content gap concerned PEM triggers. Sensory stimuli, orthostatic load, and positive emotional arousal were each raised by multiple participants as absent from the initial item set.

*“Important triggers that I find missing: sensory stimuli — these do not need to reach the threshold of ‘noise’; tactile or olfactory stimuli may also be involved.”* — P01, feedback questionnaire

*“Orthostatic load (e.g., standing or sitting) must be explicitly included as a trigger and assessed accordingly, if appropriate also in the scoring.”* — P03, P04, P06, joint supplementary document

*“… what is also always missing under emotional triggers is that joy can be an absolute trigger too. So — even when I feel happy […] a crash is unfortunately almost inevitable.”* — P03, interview

**Screening item, pacing, and medication**

Three participants recommended a gate-keeping screening item at the outset to confirm the presence of PEM before full questionnaire completion. Pacing and coping strategies and the influence of medication on PEM severity were each flagged by a small number of respondents.

*“Coping / unconscious pacing should be taken into account.”* — P12, supplementary document

*“For me personally, a PEM questionnaire would feel more ‘real’ if the influence of medication were considered.”* — P02, feedback questionnaire

**Irreversibility, exacerbating factors, and other gaps**

Four participants noted that potential irreversibility of PEM was not addressed. Five participants described secondary exacerbating factors — including hormonal fluctuations, disrupted circadian rhythm, intolerances, and infections — as relevant but absent. Isolated references were made to comorbidities, social support structures, and assessment of PEM in paediatric populations.

**S1.4 Linguistic Criticism**

Linguistic criticism was raised by 12 participants across 76 coded segments, addressing terminology, precision, and adequacy of symptom descriptors (Table S1.4).

| **Subcategory** | **Segments (n)** | **Participants (n)** |
| --- | --- | --- |
| **Linguistic criticism (total)** | 76 | 12 |
| Inappropriate terminology | 33 | 10 |
| Vague or imprecise wording | 26 | 10 |
| Inadequate symptom descriptors | 10 | 5 |
| Complex or contradictory wording | 7 | 6 |

*Note. Segments could be assigned to multiple subcategories.*

**Inappropriate terminology**

Ten participants identified terms such as Erschöpfung (exhaustion), Müdigkeit (fatigue), and Schwäche (weakness) as problematic, arguing that these words failed to capture the qualitatively distinct phenomenology of PEM and risked conflation with non-specific tiredness.

*“Difficult wording — beyond a certain severity, it is not ‘already exhausted’ but rather a daily sensation of having been poisoned […]”* — P09, PDF annotations

**Vague or imprecise wording**

Ten participants criticised the use of undefined quantifiers and ambiguous descriptors. Examples included terms such as ‘type of exertion’, ‘typical activity’, and ‘longer conversation’, which participants noted were open to highly divergent interpretation. Inconsistency in time and frequency references was also raised.

*“Terms need to be defined more precisely: what is a longer conversation? For one person it is 20 minutes, for another it is 2 hours.”* — P10, feedback questionnaire

*“The wording in the questionnaire is in parts vague, ambiguous, or linguistically suggestive. Examples: ‘all your symptoms’, ‘mild worsening’, ‘typical exhaustion’.”* — P03, feedback questionnaire

**Inadequate symptom descriptors and complex wording**

Five participants reported that specific response options did not adequately match their subjective symptom experience. A further six noted that certain items combined multiple aspects — such as symptom description, frequency, and duration — within a single sentence, creating ambiguity or apparent contradiction.

*“The response option ‘breathlessness or the feeling of not getting enough air’ does not correspond to my experience, even though this is fundamentally a major symptom of mine. Instead of ‘not getting enough air’, I think ‘shortness of breath’ or ‘exertion-induced and/or positional shortness of breath’ would be more appropriate […]”* — P02, feedback questionnaire

**S1.5 Structural Criticism**

Ten participants raised structural concerns across 24 coded segments, relating primarily to response format, layout, and item ordering (Table S1.5).

| **Subcategory** | **Segments (n)** | **Participants (n)** |
| --- | --- | --- |
| **Structural criticism (total)** | 24 | 10 |
| Inappropriate response format | 14 | 8 |
| Layout and formatting | 5 | 3 |
| Item or questionnaire length | 3 | 1 |
| Item ordering | 2 | 1 |

*Note. Segments could be assigned to multiple subcategories.*

**Response format**

Eight participants noted that the single-answer response format was ill-suited to capturing the multidimensional variability of PEM presentations. Insufficient differentiation between adjacent response options was also raised.

*“Is it intended that only one response option may be selected for all questions? I found this particularly difficult for questions 11 and 12.”* — P02, feedback questionnaire

**Layout, length, and ordering**

Three participants requested formatting improvements, including greater spacing between text blocks, additional paragraph breaks, and larger font size. Isolated concerns were raised regarding the length of individual items and the logical sequence of questions.

**S1.6 Positive Feedback**

Ten participants contributed 23 positively coded segments (Table S1.6). Endorsement focused on the overall coherence and structure of the questionnaire, its perceived feasibility, and appreciation of the participatory development process.

| **Subcategory** | **Segments (n)** | **Participants (n)** |
| --- | --- | --- |
| **Positive feedback (total)** | 23 | 10 |
| Coherence and structure | 14 | 6 |
| Appreciation / acknowledgement | 4 | 4 |
| Feasibility | 3 | 3 |
| Comprehensibility | 2 | 2 |

*“You are definitely on the right track. Developing a good questionnaire is difficult — thank you for seeking feedback!”* — P07, feedback questionnaire

**S1.7 Burden of Completion**

Six participants reported experiencing burden during questionnaire completion across nine coded segments (Table S1.7). Cognitive burden — associated primarily with retrospective symptom recall over the preceding weeks — was most frequently mentioned. Emotional and physical burden were each reported by fewer participants.

| **Subcategory** | **Segments (n)** | **Participants (n)** |
| --- | --- | --- |
| **Burden of completion (total)** | 9 | 6 |
| Cognitive burden | 6 | 6 |
| Emotional burden | 2 | 2 |
| Physical burden | 1 | 1 |

*Note. Emotional burden included frustration arising from the perceived conflation of PEM with general fatigue in item wording.*

**Expert Panel Qualitative Findings: VPEM Questionnaire Content Validation**

The following table summarises qualitative feedback and revision suggestions from the expert panel (N = 11), organised by evaluation dimension. Feedback was collected through open response fields accompanying each quantitative rating item. Where multiple closely related comments addressed the same topic cluster, these have been consolidated for conciseness.

**Table S2. Summary of expert panel qualitative feedback and revision suggestions by evaluation dimension (N = 11)**

| **Dimension** | **Expert feedback** | **Item(s)** |
| --- | --- | --- |
| **Content validity** | • Alternative recall periods of four and twelve weeks were proposed alongside the eight-week timeframe; earlier episodes outside the recall window were noted as potentially clinically relevant.  • Explicit capture of continuously worsening trajectories ("ongoing decliners") was recommended. | A3 / general |
|  | • Delayed symptom onset was not considered a mandatory diagnostic criterion; disproportionate symptom severity relative to the preceding exertion was identified as the essential defining feature.  • Separate assessment of the occurrence and duration of delayed onset was suggested.  • Inclusion of social stressors (e.g., conversations, social interactions) as PEM triggers was recommended. | General |
| **Comprehensibility** | • Short, clear sentence structures and a B1 reading level were recommended.  • Replacement of "cognitive" with "mental exertion" was proposed for improved lay comprehensibility.  • The PEM definition in item 1 was described as extensive; a more concise formulation was suggested.  • Consistent use of key terms across items was recommended (e.g., "sensory/environmental stimuli"; "orthostatic activity/postural changes").  • Column headers in the tables of items 3 and 4 were considered insufficiently precise and in need of revision. | B1–B3 |
| **Practicability** | • Addition of a response option "none of these descriptions apply", accompanied by a free-text field, was suggested to better accommodate individual symptom presentations not captured by existing categories. | C3 |
| **Methodological aspects** | • The boundary between delayed symptom worsening and a crash was described as insufficiently defined and in need of clarification.  • An inconsistency was noted between the minimum PEM duration criterion (≥14 hours) specified in item 1 and shorter duration options available in the crash duration response categories of item 4. | D1–D3 |
| **Target-group specificity** | • Concern was raised that severely affected individuals may find the questionnaire cognitively demanding.  • Development of a two-part format accommodating different severity grades was proposed as a potential solution. | E1 |

*Note.* Feedback is presented as received and has not been pre-filtered by implementation status. Item codes correspond to the evaluation dimensions in Tables 3 and 4 of the main manuscript. A complete tabular overview of all expert comments, proposed revisions, and implementation decisions is available from the corresponding author on request.
