# Supplementary material for: Development and initial content validation of the Vienna post-exertional malaise questionnaire: an iterative mixed-methods study
Source: Qual Life Res. 2026 Nov 7;35(12):327. doi: 10.1007/s11136-026-04434-2 (PMC13744550; doi:10.1007/s11136-026-04434-2)
Supplement: Supplementary file 3 [file 11136_2026_4434_MOESM3_ESM.docx]

**Supplementary File S2**

**Complete Revision Matrices: Triangulation Cycles 1 and 2 for the VPEM Questionnaire Development**

This file presents the complete revision matrices from both triangulation cycles of the VPEM Questionnaire development process. Tables S2.1–S2.6 correspond to Cycle 1 (target-group evaluation), and Table S2.7 summarises the decisions from Cycle 2 (expert-panel evaluation). Decision categories used across the matrices were adopted, partially adopted, not adopted, noted, and, in Cycle 2, retained. Colour coding is used for adopted (green), partially adopted (amber), not adopted (red), and noted (grey).

**S2.1 Triangulation Cycle 1: Target-Group Evaluation**

The following tables present the complete revision decisions derived from the target-group evaluation phase, organised by main qualitative category.

**Table S2.1 Conceptual revisions**

| **Subcode** | **Decision** | **Implementation / rationale** |
| --- | --- | --- |
| PEM–fatigue/exhaustion boundary | **Adopted** | PEM redefined (item 1); explicit demarcation from non-specific fatigue and exhaustion. |
| PEM–psychological symptoms boundary | **Adopted** | Psychological symptoms removed from core construct; retained only as secondary, context-dependent phenomena. |
| PEM as mechanism, not symptom list | **Adopted** | PEM conceptualised as an overarching pathophysiological mechanism of disproportionate post-exertional deterioration; symptom enumeration abandoned. |
| Inadequate symptom pattern representation | **Adopted** | Dedicated item on crash-associated additional symptoms introduced (item 5). |
| Stigmatisation risk | **Adopted** | Stigmatising terms and attributions removed throughout. |
| Heterogeneity / variability of PEM | **Partially adopted** | Severity-graded exertion matrix (item 2) and differentiated crash assessment by severity (item 3) and recovery duration (item 4) introduced. Full coverage of day-to-day fluctuations not implemented for practicability reasons. |
| Temporal characteristics (latency / duration) | **Partially adopted** | Delayed onset incorporated into definition (item 1); minimum crash duration of 14 hours specified; crash frequency (item 3) and recovery duration (item 4) systematically assessed. Explicit quantification of exact latency not implemented due to retrospective recall limitations. |
| Unclear rating scale / scoring | **Noted – complete redesign** | Scoring system fundamentally reconceived; non-linear weighting introduced. |
| Redundancy / content overlap | **Noted – major revision** | Overlapping items consolidated; item count reduced from 13 to 5. |

*Note.* "Adopted" indicates direct implementation of the feedback; "Noted – reconception/major revision" indicates that the feedback informed a broader structural or conceptual redesign rather than item-level modification.

**Table S2.2 Content additions and exclusions**

| **Subcode** | **Decision** | **Implementation / rationale** |
| --- | --- | --- |
| PEM triggers: sensory stimuli | **Adopted** | Added to exertion matrix (item 2) as "environmental stimuli". |
| PEM triggers: orthostatic load | **Adopted** | Incorporated as a distinct trigger category in the exertion matrix (item 2). |
| PEM triggers: positive emotional arousal | **Adopted** | Included as examples within the emotional/social trigger category (item 2). |
| PEM irreversibility | **Adopted** | New item added (item 4) and integrated into scoring. |
| Coping strategies / pacing | **Adopted** | Non-scored contextualisation item on pacing use added as prefatory question. |
| Screening item (PEM yes/no) | **Not adopted** | Risk of false-negative self-classification, particularly in individuals with uncertain symptom attribution, would reduce diagnostic sensitivity. |
| Medication effects on PEM | **Not adopted** | External moderating factor; not a constituent feature of PEM itself. |
| Exacerbating / associated factors | **Not adopted** | Outside primary construct scope. |
| Additional symptoms (general) | **Not adopted** | Symptom-centred extension not consistent with reconceptualised construct approach. |
| Comorbidities | **Not adopted** | Requires independent assessment instrument. |
| Social support / security | **Not adopted** | Outside primary construct scope. |
| PEM in children and adolescents | **Not adopted** | Requires separate, age-adapted instrument development. |

*Note.* Items not adopted were excluded on grounds of construct scope, instrument economy, or methodological risk as specified in the rationale column.

**Table S2.3 Linguistic revisions**

| **Subcode** | **Decision** | **Implementation / rationale** |
| --- | --- | --- |
| Inappropriate terminology | **Adopted** | Terms identified as inadequate (e.g., Erschöpfung, Müdigkeit, Schwäche) removed or replaced with construct-appropriate language. |
| Complex / contradictory wording | **Adopted** | Multi-aspect sentences simplified; contradictory phrasing resolved. |
| Vague / imprecise wording | **Adopted** | Time and frequency references standardised (hours, days, weeks); vague quantifiers replaced with anchored examples. |
| Inadequate symptom descriptors | **Noted – reconception** | Isolated single-symptom response options replaced through comprehensive reconceptualisation of item structure. |

*Note.* "Noted – reconception" indicates that the feedback was addressed through structural redesign rather than direct item-level language revision.

**Table S2.4 Structural revisions**

| **Subcode** | **Decision** | **Implementation / rationale** |
| --- | --- | --- |
| Inappropriate response format | **Partially adopted** | Response options sharpened and gradations clarified. Structured multiple-count items introduced for crash severity and recovery duration. Classical multiple-answer formats not adopted to preserve response comparability. |
| Layout and formatting | **Noted – major revision** | Readability and visual structure improved; each item presented on a separate page with increased spacing. |
| Item ordering | **Noted – major revision** | Item sequence reorganised as part of overall structural revision. |
| Item / questionnaire length | **Partially adopted** | Item count reduced from 13 to 5 core questions. Standard-format sub-scales retained in full to preserve content precision. |

*Note.* "Noted – major revision" indicates that the feedback was addressed within the overall structural reorganisation of the instrument.

**Table S2.5 Burden of completion**

| **Subcode** | **Role in revision** | **Interpretation** |
| --- | --- | --- |
| Cognitive burden | Noted – interpretation | Inherent trade-off between content precision and completion economy; item depth not reduced. |
| Emotional burden | Indirectly addressed | Conceptual ambiguities and frustrating formulations resolved through linguistic and conceptual revision. |
| Physical burden | Noted – interpretation | No structural modification; addressed through overall item reduction. |

*Note.* Burden-related feedback did not directly drive item revision but informed interpretation of findings and contextualised the overall development approach.

**Table S2.6 Positive feedback**

| **Subcode** | **Role in revision** | **Interpretation** |
| --- | --- | --- |
| Coherence and structure | Validation support | Confirms instrument coherence; aligns with high practicability ratings (F6: 92.3%, F7: 61.5%). |
| Appreciation / acknowledgement | Contextualising | Reflects acceptance of the participatory development process. |
| Feasibility | Validation support | Corroborates quantitative practicability ratings. |
| Comprehensibility | Validation support | Supports quantitative comprehensibility ratings (F5: 76.9%). |

*Note.* Positive feedback was not used to guide revision decisions but was documented as evidence of instrument acceptability and face validity.

**S2.2 Triangulation Cycle 2: Expert Panel Evaluation**

Table S2.7 summarises all revision decisions derived from the expert panel evaluation phase, including quantitative rating context, qualitative feedback, decision, and implementation rationale.

**Table S2.7 Expert panel revision decisions by dimension**

| **Dimension / item** | **Expert feedback** | **Decision** | **Implementation / rationale** |
| --- | --- | --- | --- |
| Content validity A1–A3 | Eight-week recall period rated acceptable to very suitable (63.7% categories 3–5). Alternatives of 4 and 12 weeks proposed. Earlier episodes and "ongoing decliners" noted as not captured. | **Retained** | Eight-week window retained; shorter periods risk sampling a single phase; longer periods increase recall bias. Ongoing decline acknowledged as a limitation. |
| Content validity General | Delayed onset not considered mandatory; disproportionality to exertion identified as the essential defining feature. | **Adopted** | Delayed onset reformulated as frequent but non-obligatory; disproportionality foregrounded in definition. |
| Content validity Item 2 | Social stressors recommended for inclusion as PEM triggers. | **Adopted** | Social dimension consolidated with emotional triggers (emotional/social category) in exertion matrix. |
| Comprehensibility B1–B3 | B1 reading level and short sentences recommended. "Cognitive" → "mental exertion" proposed. PEM definition described as extensive. Consistent terminology recommended. Column headers in items 3–4 considered imprecise. | **Partially adopted** | Plain-language equivalents used throughout. "Cognitive" retained but supplemented with "mental activity". Definition maintained in full scope. Terminology standardised. Column headers revised for precision. |
| Practicability C1–C3 | Addition of "none of the above" with free-text field proposed. | **Not adopted** | Free-text format would reduce standardisation and increase respondent burden; response categories confirmed as well differentiated (C3: 90.9%). |
| Methodological aspects D1–D3 | Boundary between delayed worsening and crash described as unclear. Inconsistency between ≥14-hour minimum duration (item 1) and shorter options in item 4 noted. | **Adopted** | "Crash" adopted consistently throughout. Fixed minimum duration removed from definition; inconsistency resolved. |
| Target-group specificity E1 | Severely affected individuals may be overwhelmed; two-part instrument proposed. | **Noted – not adopted** | Requires separate development and validation pathway; outside scope of present study. |

*Note.* All expert panel methodological items (D1–D3) received exclusively favourable ratings (100% categories 4–5) and required no structural modification. Items on medication effects and comorbidities were not raised by the expert panel and were confirmed as appropriately excluded from the core instrument.
