# Supplementary material for: Development and initial content validation of the Vienna post-exertional malaise questionnaire: an iterative mixed-methods study
Source: Qual Life Res. 2026 Nov 7;35(12):327. doi: 10.1007/s11136-026-04434-2 (PMC13744550; doi:10.1007/s11136-026-04434-2)
Supplement: Supplementary file 4 [file 11136_2026_4434_MOESM4_ESM.docx]

**Supplementary File S3**

**Final Vienna Post-Exertional Malaise Questionnaire (VPEM)**

**English Version**

**Instructions for Participants**

When answering, please think about a typical day in the last 8 weeks, not a particularly good or bad day.

**Question 1: Crashes After Exertion**

What is a crash? Your health condition deteriorates significantly after physical, mental or emotional exertion.

**Key characteristics:**

- The deterioration usually occurs after a delay, but sometimes immediately.
- Rest or sleep does not lead to full recovery.

**Examples of exertion:**

- Physical: sports, housework, climbing stairs, normal daily tasks
- Mental: concentration, difficult tasks
- Emotional/social: stress, demanding conversations, strong emotions (e.g., joy, anger), receiving visitors, meeting many people
- Environmental stimuli: bright light, loud noise, strong smells (often even normal stimuli are sufficient)
- Orthostatic (position changes): standing, sitting upright (even briefly), changing position – as in Postural Orthostatic Tachycardia Syndrome (POTS)

**Question: How often did you experience such a crash in the last 8 weeks? Please select one answer:**

☐ Continuously – new deterioration begins before the previous crash has ended

☐ Almost daily – 4–7 days per week

☐ Several times per week – 2–3 times per week

☐ About weekly – approximately once per week

☐ Rarely – 1–4 times in the last 8 weeks

☐ Never – no such reactions

**Question 2: When Do You Experience a Crash?**

*Note: The examples show typical everyday situations. These may vary from person to person. For some, a family celebration is less strenuous than a doctor’s visit or an argument; for others, it is the opposite.*

**Question: Thinking about the last 8 weeks, please indicate the level of exertion from which you were highly likely to experience a crash. For each type of exertion, select only one level that best describes your situation.**

| **Type of exertion** | **At minimal exertion** | **At very low exertion** | **At low exertion** | **At moderate exertion** | **At greater exertion** | **At very high exertion** | **No reaction** |
| --- | --- | --- | --- | --- | --- | --- | --- |
| **Physical activity** | ☐ Basic personal care (dressing, washing, using the toilet) | ☐ Walking a few steps, standing for 2–5 minutes | ☐ Showering while seated, light housework (approx. 5–15 minutes) | ☐ Short walk on level ground, light household activities (approx. 15–30 minutes) | ☐ Longer walk, more demanding housework (approx. 30–60 minutes), carrying groceries home (approx. 10–15 minutes) | ☐ Sustained vigorous physical activity (approx. ≥30 minutes), such as hiking, cycling, jogging, strenuous gardening, or recreational sports | ☐ No deterioration |
| **Mental/cognitive activity** | ☐ Reading short texts, watching a brief TV sequence | ☐ Short conversations, text messages, 5-minute phone call | ☐ Reading a book, longer conversation, writing an email | ☐ Filling out forms, focused cognitive work (approx. 30 minutes) | ☐ Extended cognitive work (from approx. 2 hours) | ☐ Full workday with sustained mental/cognitive demands | ☐ No deterioration |
| **Environmental stimuli (e.g., light, noise)** | ☐ Dimmed light, very quiet environment | ☐ Normal daylight, quiet environment | ☐ Bright rooms, typical everyday noises | ☐ Outdoors without protection (e.g., without sunglasses or ear protection), moderate stimulus exposure | ☐ Loud environment, e.g., supermarket or café | ☐ Concert, cinema or party with many stimuli | ☐ No deterioration |
| **Emotional/social stimuli** | ☐ Small surprise, slight anticipation | ☐ Brief friendly interaction, short message exchange | ☐ Receiving a visitor, short appointment | ☐ Doctor’s visit, dealing with authorities, minor conflict | ☐ Important or emotionally intense conversation, distressing news | ☐ Argument/conflict conversation, family celebration or large event | ☐ No deterioration |
| **Positional changes** | ☐ Sitting up in bed or remaining upright in bed for a few minutes | ☐ Sitting on edge of bed (less than 10 minutes) | ☐ Sitting on a chair (more than 10 minutes) | ☐ Standing briefly, performing simple tasks (5 minutes) | ☐ Standing or walking for about 15–30 minutes | ☐ Standing or walking for more than 30 minutes | ☐ No deterioration |

**Question 3: How Severe Were Your Crashes?**

*Note: Enter how many crashes you had at each severity level. Please complete only the rows that apply to your crashes; all other rows may be left blank.*

**Examples:**

- You had 3 crashes: 1 mild, 1 severe, 1 very severe → enter “1” under “mild”, “severe”, and “very severe”.
- You had 3 crashes: 2 mild, 1 severe → enter “2” under “mild” and “1” under “severe”.

**Question: Think about the last 8 weeks. How limited were you in daily life during a crash?**

| **Number of crashes** | **Severity of crash** | **Description** |
| --- | --- | --- |
|  | **Very mild limitations** | More breaks needed, but activities still possible |
|  | **Mild limitations** | Some things slower or more difficult; minor restrictions in daily life |
|  | **Moderate limitations** | Some activities sometimes not possible (e.g., cooking, reading, conversations) |
|  | **Severe limitations** | Many activities not possible; even meeting basic needs is difficult |
|  | **Very severe limitations** | Mostly in bed; hardly any stimuli or conversations possible; basic care severely impaired |
|  | **Almost complete limitation** | Even eating, drinking, or using the toilet is only possible with help or great effort |

**Question 4: How Long Does a Crash Last? (Time until symptoms disappear)**

**Note:**

- The total number of crashes should match your answer in Question 3.
- Please indicate how long the crash lasts when you rest as completely as possible afterwards — with plenty of rest, reduced activity, and no additional stimuli.
- Duration refers only to how long the symptoms persist; even a short symptom duration still qualifies as a crash if post-exertional worsening occurred.

**Question: Please think about the crashes in the last 8 weeks. How long did it usually take until your symptoms disappeared — meaning until you felt as you did before the crash?**

| **Number of crashes** | **Duration** |
| --- | --- |
|  | Symptoms disappear within 1–3 hours |
|  | Symptoms disappear within 4–13 hours |
|  | Symptoms last about 14–24 hours (e.g., until the next day) |
|  | Symptoms last 1–3 days |
|  | Symptoms last 4–7 days |
|  | Symptoms last 1–4 weeks |
|  | No full recovery: after a crash, I have not regained my previous functional level or have worsened permanently |

**Question 5: New Symptoms During a Crash**

*Note: This question is about whether you sometimes experience additional symptoms during a crash that you normally do not have. Examples include fever that you do not usually have, or pain in areas where you do not usually experience pain. These symptoms disappear once the crash ends.*

**Question: How often did you experience such new symptoms during a crash in the last 8 weeks?**

| **Frequency** | **Description** |
| --- | --- |
| ☐ Never | No new symptoms |
| ☐ Very rarely | In <10% of crashes |
| ☐ Rarely | In about 10–25% of crashes |
| ☐ Sometimes | In about 25–50% of crashes |
| ☐ Frequently | In about 50–75% of crashes |
| ☐ Very frequently | In about 75–90% of crashes |
| ☐ Always | In all crashes |

**Question 6: Pacing**

*Note: Pacing means managing your energy and taking breaks to avoid overexertion and prevent further deterioration of your health.*

**Question: Do you use pacing to avoid crashes? Please select one answer.**

☐ Yes, daily and consistently.
☐ Yes, partially, for example only for physical activities.
☐ Occasionally – I use pacing, but not regularly.
☐ Hardly ever – I would like to use pacing but can implement it only to a very limited extent because of external circumstances, such as caregiving responsibilities, children, or lack of support.
☐ No – I am familiar with pacing but choose not to use it.
☐ No – I am not familiar with pacing.

**Question 7: How Well Does Pacing Work for You?**

Question: How well can you avoid crashes through pacing? Please select one answer.

☐ Very well — I have no or almost no crashes anymore as a result

☐ Well — I have significantly fewer crashes as a result

☐ Moderately — it helps somewhat, but I still have regular crashes

☐ Hardly at all — I still have frequent crashes

☐ Not at all — I have just as many crashes as before, despite pacing

**German Version**

**Instruktion für die Teilnehmer:innen**

Bitte denken Sie bei Ihren Antworten an einen normalen Tag in den letzten 8 Wochen – nicht an einen besonders guten oder besonders schlechten Tag.

**Frage 1: Crashs nach Belastung**

Was ist ein Crash? Ihr Gesundheitszustand verschlechtert sich deutlich nach körperlicher, geistiger oder emotionaler Anstrengung.

**Wichtige Merkmale:**

- Die Verschlechterung tritt meist zeitverzögert auf, manchmal aber auch sofort.
- Ruhe oder Schlaf führen nicht zur vollständigen Erholung.

**Beispiele für Belastungen:**

- Körperlich: Sport, Hausarbeit, Treppen steigen, normale Alltagsaufgaben
- Geistig: Konzentration, schwierige Aufgaben
- Emotional/Sozial: Stress, anstrengende Gespräche, starke Gefühle (z. B. Freude, Wut), Besuche bekommen, viele Menschen treffen
- Umweltreize: Helles Licht, laute Geräusche, starke Gerüche (oft reichen schon normale Reize)
- Orthostatisch (Lagewechsel): Stehen, aufrechtes Sitzen (auch kurz), Positionswechsel – wie beim Posturalen Tachykardie-Syndrom (POTS)

**Frage: Wie oft hatten Sie in den letzten 8 Wochen einen solchen Crash? Bitte wählen Sie nur eine Antwort aus:**

☐ Durchgehend – Neue Verschlechterung beginnt, bevor die vorherige ganz vorbei ist

☐ Fast täglich – an 4–7 Tagen pro Woche

☐ Mehrmals wöchentlich – 2–3-mal pro Woche

☐ Etwa wöchentlich – etwa 1-mal pro Woche

☐ Selten – 1–4-mal in den letzten 8 Wochen

☐ Nie – keine solchen Reaktionen

**Frage 2: Wann kommt es bei Ihnen zu einem Crash?**

*Hinweise: Die Beispiele zeigen typische Alltagssituationen. Sie können sich von Person zu Person unterscheiden. Für manche ist eine Familienfeier weniger anstrengend als ein Arztbesuch oder ein Streitgespräch – für andere ist es umgekehrt.*

**Frage: Bitte geben Sie für die letzten 8 Wochen an, ab welcher Belastungsstufe es bei Ihnen mit großer Wahrscheinlichkeit zu einem Crash kommt. Kreuzen Sie bei jeder Art von Belastung nur eine Stufe an, die Ihre Situation am besten beschreibt.**

| **Belastungsart** | **Bei allerkleinster Belastung** | **Bei sehr geringer Belastung** | **Bei geringer Belastung** | **Bei moderater Belastung** | **Bei größerer Belastung** | **Bei sehr großer Belastung** | **Keine Reaktion** |
| --- | --- | --- | --- | --- | --- | --- | --- |
| **Körperliche Aktivität** | ☐ Einfache Körperpflege (Anziehen, Waschen, Toilettengang | ☐ Wenige Schritte gehen, 2–5 Minuten stehen | ☐ Duschen im Sitzen, leichte Hausarbeit (ca. 5–15 Minuten) | ☐ Kurzer Spaziergang auf ebener Strecke, leichte Haushaltstätigkeiten (ca. 15–30 Minuten) | ☐ Längerer Spaziergang (ca. 30–60 Minuten), Einkäufe nach Hause tragen (ca. 10–15 Minuten) oder anstrengendere Hausarbeit (ca. 30–60 Minuten) | ☐ Länger andauernde intensive körperliche Aktivität (ca. ≥30 Minuten), z. B. Wandern, Radfahren, Joggen, anstrengende Gartenarbeit oder Freizeitsport | ☐ Keine Verschlechterung |
| **Geistige/Kognitive Aktivität** | ☐ Kurze Texte lesen, kurze TV-Sequenz | ☐ Kurze Gespräche, SMS, 5-Minuten-Telefonat | ☐ Buch lesen, längeres Gespräch, E-Mail schreiben | ☐ Formulare ausfüllen, konzentrierte kognitive Arbeit (ca. 30 Minuten) | ☐ Längere kognitive Arbeit (ab ca. 2 Stunden) | ☐ Ganzer Arbeitstag mit anhaltender geistiger/kognitiver Beanspruchung | ☐ Keine Verschlechterung |
| **Umweltreize (z. B. Licht, Geräusche)** | ☐ Gedämpftes Licht, sehr ruhige Umgebung | ☐ Normales Tageslicht, ruhige Umgebung | ☐ Helle Räume, übliche Alltagsgeräusche | ☐ Aufenthalt im Freien ohne Schutz (z. B. ohne Sonnenbrille/Ohrschutz), moderate Reizbelastung | ☐ Laute Umgebung, z. B. Supermarkt oder Café | ☐ Konzert, Kino oder Party mit vielen Reizen | ☐ Keine Verschlechterung |
| **Emotionale/Soziale Reize** | ☐ Kleine Überraschung, leichte Vorfreude | ☐ Kurze freundliche Interaktion, kurzer Nachrichtenaustausch | ☐ Besuch empfangen, kurzer Termin | ☐ Arztbesuch, Behördengang, kleiner Konflikt | ☐ Wichtiges oder emotional intensives Gespräch, belastende Nachricht | ☐ Streitgespräch, Familienfeier oder große Veranstaltung | ☐ Keine Verschlechterung |
| **Lageänderung/Positionswechsel** | ☐ Schon beim Aufrichten im Bett oder bei aufrechtem Sitzen im Bett für wenige Minuten | ☐ Am Bettrand sitzen (weniger als 10 Minuten) | ☐ Auf einem Stuhl sitzen (mehr als 10 Minuten) | ☐ Kurz stehen, einfache Tätigkeiten ausführen (5 Minuten) | ☐ Stehen oder Gehen für etwa 15–30 Minuten | ☐ Stehen oder Gehen für mehr als 30 Minuten | ☐ Keine Verschlechterung |

**Frage 3: Wie schwer waren Ihre Crashs?**

*Hinweis: Tragen Sie ein, wie oft Sie Crashs in der jeweiligen Schwere hatten. Bitte füllen Sie nur die Zeilen aus, die auf Ihre Crashs zutreffen; alle anderen Zeilen können leer bleiben.*

**Beispiele:**

- Sie hatten 3 Crashs: 1× leicht, 1× stark, 1× sehr stark → Tragen Sie bei „leicht“, „stark“ und „sehr stark“ jeweils eine 1 ein.
- Sie hatten 3 Crashs: 2× leicht, 1× stark → Tragen Sie bei „leicht“ eine 2 und bei „stark“ eine 1 ein.

**Frage: Denken Sie an die letzten 8 Wochen. Wie stark waren Sie während eines Crashs im Alltag eingeschränkt?**

| **Anzahl der Crashs** | **Schwere des Crashs** | **Beschreibung** |
| --- | --- | --- |
|  | **Sehr leichte Einschränkungen** | Mehr Pausen nötig, Aktivitäten aber weiterhin gut möglich |
|  | **Leichte Einschränkungen** | Einige Dinge langsamer oder schwerer möglich, kleine Einschränkungen im Alltag |
|  | **Mäßige Einschränkungen** | Einige Tätigkeiten manchmal nicht möglich (z. B. Kochen, Lesen, Gespräche) |
|  | **Starke Einschränkungen** | Viele Tätigkeiten nicht mehr möglich; selbst die Erledigung von Grundbedürfnissen ist schwierig |
|  | **Sehr starke Einschränkungen** | Meist im Bett, kaum Reize oder Gespräche möglich; Grundversorgung stark erschwert |
|  | **Fast vollständige Einschränkung** | Selbst Essen, Trinken oder Toilettengang nur noch mit Hilfe oder mit großer Anstrengung möglich |

**Frage 4: Wie lange dauert ein Crash? (Zeit, bis die Beschwerden wieder verschwinden)**

**Hinweis:**

- Die Gesamtanzahl der Crashs sollte mit Ihrer Antwort bei Frage 3 übereinstimmen.
- Bitte geben Sie an, wie lange der Crash dauert, wenn Sie sich danach möglichst vollständig ausruhen – also mit viel Ruhe, Rückzug und ohne zusätzliche Reize.
- Die Dauer bezieht sich nur darauf, wie lange die Beschwerden anhalten; auch eine kurze Beschwerdedauer zählt als Crash, wenn es zu einer Verschlechterung nach Belastung gekommen ist.

**Frage: Bitte denken Sie an die Crashs in den letzten 8 Wochen. Wie lange hat es normalerweise gedauert, bis Ihre Beschwerden wieder verschwunden waren – also bis Sie sich wieder so gefühlt haben wie vor dem Crash?**

| **Anzahl der Crashs** | **Dauer** |
| --- | --- |
|  | Die Beschwerden verschwinden innerhalb von 1–3 Stunden |
|  | Die Beschwerden verschwinden innerhalb von 4–13 Stunden |
|  | Die Beschwerden dauern etwa 14–24 Stunden (z. B. bis zum nächsten Tag) |
|  | Die Beschwerden dauern 1–3 Tage |
|  | Die Beschwerden dauern 4–7 Tage |
|  | Die Beschwerden dauern 1–4 Wochen |
|  | Keine vollständige Erholung: Nach einem Crash habe ich mein vorheriges Funktionsniveau nicht wieder erreicht oder mein Zustand hat sich dauerhaft verschlechtert. |

**Frage 5: Neue Symptome während eines Crashs**

*Hinweis: Bei dieser Frage geht es darum, ob während eines Crashs manchmal zusätzliche Symptome auftreten, die Sie normalerweise nicht haben. Beispiele dafür sind Fieber, das Sie sonst nicht haben, oder Schmerzen an Körperstellen, an denen Sie sonst keine Schmerzen haben. Diese Symptome verschwinden wieder, sobald der Crash vorbei ist.*

**Frage: Wie oft sind bei Ihnen in den letzten 8 Wochen während eines Crashs solche neuen Symptome aufgetreten?**

| **Häufigkeit** | **Beschreibung** |
| --- | --- |
| ☐ Nie | Keine neuen Symptome |
| ☐ Sehr selten | Bei weniger als 10 % meiner Crashs |
| ☐ Selten | Bei etwa 10–25 % meiner Crashs |
| ☐ Manchmal | Bei etwa 25–50 % meiner Crashs |
| ☐ Häufig | Bei etwa 50–75 % meiner Crashs |
| ☐ Sehr häufig | Bei etwa 75–90 % meiner Crashs |
| ☐ Immer | Bei allen meinen Crashs |

**Frage 6: Pacing**

*Hinweis: Pacing bedeutet: Sie teilen Ihre Kraft gut ein und machen genug Pausen. So vermeiden Sie eine Überlastung, damit sich Ihr Gesundheitszustand nicht weiter verschlechtert.*

**Frage: Wenden Sie Pacing an, um Crashs zu vermeiden? Bitte wählen Sie nur eine Antwort aus.**

☐ Ja, täglich und konsequent

☐ Ja, teilweise (z. B. nur bei körperlichen Aktivitäten)

☐ Nur gelegentlich– ich wende Pacing an, aber nicht regelmäßig

☐ Kaum – ich würde gerne Pacing anwenden, kann es aber wegen äußerer Umstände nur sehr eingeschränkt umsetzen (z. B. wegen Pflege, Kindern oder fehlender Hilfe)

☐ Nein – ich kenne Pacing, wende es aber nicht an

☐ Nein – ich kenne Pacing nicht

**Frage 7: Wie gut funktioniert Pacing bei Ihnen?**

**Frage: Wie gut können Sie durch Pacing Crashs vermeiden? Bitte wählen Sie nur eine Antwort aus.**

☐ Sehr gut – ich habe dadurch keine oder fast keine Crashs mehr

☐ Gut – ich habe dadurch deutlich weniger Crashs

☐ Mittelmäßig – es hilft etwas, aber ich habe noch regelmäßig Crashs

☐ Kaum – ich habe trotzdem häufig Crashs

☐ Gar nicht – ich habe trotz Pacing genauso viele Crashs wie vorher

**Provisional scoring framework and interpretation**

The VPEM Questionnaire yields a multidimensional PEM profile and, in addition, a provisional aggregate score intended for subsequent psychometric evaluation. All scored components are coded so that higher values indicate greater PEM-related burden. Questions 3 and 4 are scored as frequency-weighted mean category scores: the reported crash counts serve only to weight the category values and are not added to the score themselves, so these components remain within a fixed range irrespective of the absolute number of crashes reported.

**Item-level scoring**

| **Item** | **Response categories and assigned values** | **Range** |
| --- | --- | --- |
| Q1 — Crash frequency | never = 0; rarely (1–4 crashes in 8 weeks) = 1; about weekly = 2; several times per week = 3; almost daily = 4; continuously = 5 | 0–5 |
| Q2a–Q2e — Exertion thresholds (five trigger domains, reverse-scored) | no deterioration = 0; at very high exertion = 1; at greater = 2; at moderate = 3; at low = 4; at very low = 5; at minimal exertion = 6 | 0–6 per domain |
| Q3 — Crash severity | very mild = 1; mild = 2; moderate = 3; severe = 4; very severe = 5; almost complete limitation = 6 (0 = no crashes) | 0–6 |
| Q4 — Recovery burden | 1–3 h = 1; 4–13 h = 2; 14–24 h = 3; 1–3 days = 4; 4–7 days = 5; 1–4 weeks = 6; no full recovery = 7 (0 = no crashes) | 0–7 |
| Q5 — New crash-associated symptoms | never = 0; very rarely (<10% of crashes) = 1; rarely (10–25%) = 2; sometimes (25–50%) = 3; frequently (50–75%) = 4; very frequently (75–90%) = 5; always = 6 | 0–6 |
| Q6, Q7 — Pacing | not scored; interpreted separately as contextual variables | — |

**Frequency-weighted scoring of Questions 3 and 4**

For each item, the number of crashes reported in a category is multiplied by the category value, summed across categories, and divided by the total number of crashes reported in that item:

*Q3 or Q4 = Σ (number of crashes in category × category value) / total number of crashes*

Because “no full recovery” denotes failure to return to approximately the level immediately before a crash rather than a finite recovery duration, Question 4 is interpreted as a recovery-burden score rather than as a mean recovery duration. The “no full recovery” category retains its assigned value of 7 in the frequency-weighted Q4 calculation. For example, if three crashes are reported in this category, they contribute 3 × 7 to the numerator. In addition, a separate binary non-recovery indicator is reported (1 if at least one crash without full recovery is reported, 0 otherwise). This binary indicator does not replace or alter the crash count used in the Q4 calculation. No additional weighting beyond the assigned category value of 7 is applied.

**Aggregate score**

*VPEM total score = Q1 + Q2a + Q2b + Q2c + Q2d + Q2e + Q3 + Q4 + Q5*

For responses permitting calculation of all components, the nominal score range is 0–54, with higher values indicating greater PEM-related burden. Questions 3 and 4 may take non-integer values; their unrounded values are used to calculate the total score, which may subsequently be rounded to two decimal places for reporting. Pacing (Questions 6 and 7) is excluded, because a low crash frequency may reflect either mild disease or successful exertion avoidance.

**Worked example**

A respondent reports crashes about once a week (Q1 = 2) and indicates that crashes are highly likely at greater exertion in all five domains (Q2a–Q2e = 2 each; 10 points). In Question 3, three crashes are reported with moderate limitations (value 3) and four with severe limitations (value 4): Q3 = [(3 × 3) + (4 × 4)] / 7 = 25 / 7 = 3.57. In Question 4, the same seven crashes are distributed as two lasting 1–3 days (value 4), four lasting 4–7 days (value 5), and one involving no full recovery (value 7): Q4 = [(2 × 4) + (4 × 5) + (1 × 7)] / 7 = 35 / 7 = 5.00. Because at least one crash involved no full recovery, the binary non-recovery indicator is coded as 1. New symptoms occur during approximately 10–25% of crashes (Q5 = 2). The total score is 2 + 10 + 3.57 + 5.00 + 2 = 22.57 out of 54.

**Consistency requirements and non-evaluable responses**

Before calculation, entries in Questions 3 and 4 must be non-negative integers, and the total number of crashes reported in Question 3 must equal the total reported in Question 4. Both totals should also be broadly compatible with the crash-frequency category selected in Question 1. Because Question 1 partly refers to crash frequency over time, whereas Questions 3 and 4 count distinguishable crash episodes, clear discrepancies are reviewed as indicators of potential non-evaluability rather than assessed using rigid numerical cut-offs. Raw crash counts are used only to weight the category distributions and therefore cannot increase Questions 3 or 4 beyond their defined ranges. Incomplete or internally inconsistent responses are classified as non-evaluable; no aggregate score is calculated, and no maximum value is imputed.

**Special rules**

If no crashes are reported and Question 1 is answered “never”, Questions 3 and 4 are assigned a value of 0. The value 0 therefore denotes the absence of crashes and is a structural category rather than a severity level; where crashes are present, category values begin at 1. If Question 1 is answered “continuously” and no distinguishable episodes are reported, Questions 3 and 4 cannot be calculated. Such responses are classified as a continuous, non-episodic course and reported separately; an aggregate score is not computed and a maximum value is not imputed, as this would not be methodologically justified. Because Question 1 contributes its maximum value only when “continuously” is selected, the upper end of the nominal range is reached only where distinguishable episodes are nevertheless reported.

**Measurement assumptions**

This scoring framework is provisional and documents the intended scoring approach at the instrument-development stage. Questions 3 and 4 are calculated as frequency-weighted mean category scores, whereas the aggregate VPEM score is the unweighted sum of the component scores. Because the components have different score ranges and Question 2 contributes five separate domain scores, their potential contributions to the aggregate score are not equal. The framework uses consecutive integer coding for ordered response categories. Severity and recovery duration are summarised separately because the questionnaire does not link the severity of an individual crash to its recovery duration. The dimensional structure of the questionnaire, the suitability of combining the component scores into an aggregate score, their relative contributions, and score interpretation require psychometric evaluation.
