# Supplementary material for: Development and initial content validation of the Vienna post-exertional malaise questionnaire: an iterative mixed-methods study
Source: Qual Life Res. 2026 Nov 7;35(12):327. doi: 10.1007/s11136-026-04434-2 (PMC13744550; doi:10.1007/s11136-026-04434-2)
Supplement: Supplementary file 5 [file 11136_2026_4434_MOESM5_ESM.docx]

**Supplementary File S4**

*Initial Draft Questionnaire — German original (Part 1) and English translation (Part 2)*

This file contains the initial 13-item draft questionnaire that preceded the Vienna Post-Exertional Malaise (VPEM) Questionnaire. Part 1 reproduces the original German draft administered to participants; Part 2 provides an English translation of the same 13-item draft, supplied for transparency of the development progression. The final English and German versions of the VPEM Questionnaire are provided in Supplementary File S3.

**PART 1 | Initial Draft Questionnaire (13 items, German)**

**Frage 1: Welche der folgenden Symptome erleben Sie seit Ihrer Erkrankung häufig und über die meiste Zeit?**

*Bitte alle zutreffenden Symptome ankreuzen.*

☐ Symptomverschlechterung nach Belastung: Nach körperlicher, geistiger oder emotionaler Anstrengung treten eine deutliche Verschlechterung der Symptome sowie eine verlängerte Erholungszeit auf.

☐ Ständige Erschöpfung: Anhaltende körperliche und/oder geistige Müdigkeit, die unabhängig von der Tätigkeit auftritt und den Alltag einschränkt.

☐ Muskelschmerzen oder Brennen

☐ Kopfschmerzen oder Schwindel

☐ Übelkeit, Bauchschmerzen oder Verdauungsprobleme

☐ Grippeähnliche Beschwerden (z. B. Halsschmerzen, Fieber, Gliederschmerzen)

☐ Konzentrationsprobleme (Brain Fog)

☐ Gedächtnisprobleme

☐ Empfindlichkeit gegenüber Licht

☐ Empfindlichkeit gegenüber Lärm

☐ Kreislaufprobleme oder Schwierigkeiten beim längeren Stehen

☐ Unverträglichkeit oder schlechtere Verträglichkeit bestimmter Nahrungsmittel

☐ Nicht erholsamer Schlaf oder Schlafstörungen

☐ Glieder- oder Gelenkschmerzen

☐ Atemnot oder das Gefühl, nicht genug Luft zu bekommen

☐ Herzklopfen oder unregelmäßiger Herzschlag

☐ Empfindlichkeit gegenüber Temperaturveränderungen (z. B. Hitze/Kälte)

☐ Niedergeschlagene Stimmung oder erhöhte Reizbarkeit

☐ Andere Symptome: ____________________________

**Frage 2: Wie stark sind Sie im Vergleich zu Ihrer Zeit vor der Erkrankung in Ihren täglichen Aktivitäten eingeschränkt?**

☐ 0: Gar nicht eingeschränkt

☐ 1: Leicht eingeschränkt (gelegentliche Anpassungen nötig)

☐ 2: Moderat eingeschränkt (regelmäßige Anpassungen nötig, z. B. Pausen)

☐ 3: Stark eingeschränkt (viele Aktivitäten nicht mehr möglich)

☐ 4: Extrem eingeschränkt (nahezu alle Aktivitäten sind unmöglich)

**Frage 3: Empfinden Sie die meiste Zeit eine dauerhafte Erschöpfung oder Schwäche, die unabhängig von körperlichen, geistigen oder emotionalen Aktivitäten auftritt?**

☐ 1. Ja, fast immer.

☐ 2. Ja, häufig, aber nicht ständig.

☐ 3. Nein, nur gelegentlich.

☐ 4. Nein, kaum oder gar nicht.

**Frage 4: Wenn Sie bereits erschöpft sind, führt eine Belastung zu einer zusätzlichen Verschlimmerung der Symptome (PEM), die sich deutlich von Ihrer üblichen Erschöpfung unterscheidet?**

*Bitte denken Sie an die vergangenen 4 Wochen.*

☐ 0 = Kein Unterschied

☐ 1 = Leicht erkennbar

☐ 2 = Mäßig erkennbar

☐ 3 = Deutlich erkennbar

☐ 4 = Extrem klarer Unterschied

**Frage 5: Treten nach körperlicher, geistiger oder emotionaler Belastung häufig eine unverhältnismäßig starke Verschlechterung Ihrer Symptome sowie eine verlängerte Erholungszeit auf?**

☐ 1. Ja, fast immer nach Belastung.

☐ 2. Ja, häufig, aber nicht immer.

☐ 3. Ja, aber nur selten.

☐ 4. Nein, Belastungen haben keine zusätzlichen Auswirkungen.

**Frage 6: Wie schnell haben Sie bei einer für Sie typischen anstrengenden Tätigkeit muskuläre Ermüdung verspürt? Wie stark hat Ihre Kraft nachgelassen?**

*Beispiele: Treppensteigen, wiederholtes Aufstehen und Hinsetzen, Tragen eines Gegenstandes. Letzte 4 Wochen.*

☐ 0 Punkte: Keine Erschöpfung, die Aktivität bleibt jederzeit gleich durchführbar.

☐ 1 Punkt: Gelegentliche leichte Erschöpfung, bei Wiederholung selten schwerer.

☐ 2 Punkte: Häufig spürbare Erschöpfung, bei häufiger Wiederholung deutlich eingeschränkt.

☐ 3 Punkte: Sehr oft starke Erschöpfung, bei Wiederholung fast immer erhebliche Schwierigkeiten.

☐ 4 Punkte: Fast immer vollständige Erschöpfung; Aktivität kann nach wenigen Wiederholungen nicht mehr durchgeführt werden.

**Frage 7: Treten Verschlechterungen Ihrer Symptome nach körperlicher Aktivität auf?**

*Beispiele: Kurzes Gehen, Treppensteigen, leichte Hausarbeit.*

☐ Nie (0): Ich bemerke keine Verschlechterung nach körperlicher Aktivität.

☐ Selten (1): Gelegentlich merke ich eine leichte Verschlechterung.

☐ Manchmal (2): Etwa die Hälfte der Zeit bemerke ich eine deutliche Verschlechterung.

☐ Oft (3): Häufig treten Verschlechterungen nach körperlicher Aktivität auf.

☐ Immer (4): Jede körperliche Aktivität führt zu einer klaren Verschlechterung.

**Frage 8: Treten Verschlechterungen Ihrer Symptome nach kognitiver Aktivität auf?**

*Beispiele: Lesen, konzentrierte Arbeit am PC, längeres Gespräch.*

☐ Nie (0): Keine Verschlechterung.

☐ Selten (1): Leichte Verschlechterung gelegentlich.

☐ Manchmal (2): Deutliche Verschlechterung etwa die Hälfte der Zeit.

☐ Oft (3): Häufig Verschlechterungen.

☐ Immer (4): Jede kognitive Aktivität führt zu Verschlechterung.

**Frage 9: Treten Verschlechterungen Ihrer Symptome nach emotionaler/mentaler Belastung auf?**

*Beispiele: Stress, Streitgespräche, emotionale Herausforderungen.*

☐ Nie (0): Keine Verschlechterung.

☐ Selten (1): Leichte Verschlechterung gelegentlich.

☐ Manchmal (2): Deutliche Verschlechterung etwa die Hälfte der Zeit.

☐ Oft (3): Häufig Verschlechterungen.

☐ Immer (4): Jede emotionale Belastung führt zu Verschlechterung.

**Frage 10: Verschlimmern sich alle Ihre Symptome nach einer Belastung?**

*z. B. körperlich, kognitiv, emotional*

☐ Ja, alle Symptome verschlechtern sich gleichermaßen.

☐ Ja, alle Symptome verschlechtern sich, aber nicht in gleichem Maße.

☐ Nein, nur einige Symptome verschlechtern sich.

☐ Nein, nur ein Symptom verschlechtert sich.

☐ Nein, kein Symptom verschlechtert sich.

**Frage 11: Wie lange hält die verschlimmerte Symptomphase in der Regel an?**

☐ Einige Stunden

☐ Weniger als 24 Stunden

☐ 24–48 Stunden

☐ Mehrere Tage

☐ Eine Woche

☐ Mehrere Wochen

☐ Kehrt nicht zum Ausgangsniveau zurück

**Frage 12: Wie gut können Sie sich von der Verschlechterung Ihrer Symptome erholen, wenn Sie vollständig zur Ruhe kommen?**

*Bitte denken Sie an die letzten 4 Wochen.*

☐ 1. Sehr gut: Nach kurzer Zeit (wenige Stunden) bin ich vollständig erholt.

☐ 2. Gut: Ich erhole mich, aber es dauert mindestens einen Tag.

☐ 3. Mäßig: Die Erholung ist nur teilweise möglich und dauert mehrere Tage.

☐ 4. Schlecht: Es gibt nur minimale Besserung, selbst nach längerer Ruhe.

☐ 5. Gar nicht: Selbst nach kompletter Ruhe verbessert sich mein Zustand kaum oder gar nicht.

**Frage 13: Gibt es zusätzliche Faktoren, die Ihre Symptome nach einer Belastung verstärken?**

*Bitte jeden Faktor separat auf einer Skala von 0 bis 4 bewerten. Letzte 4 Wochen.*

| **Faktor** | **0 Keine Wirkung** | **1 Gering** | **2 Moderat** | **3 Deutlich** | **4 Extrem** |
| --- | --- | --- | --- | --- | --- |
| Schlafmangel | ☐ | ☐ | ☐ | ☐ | ☐ |
| Stress oder emotionale Belastung | ☐ | ☐ | ☐ | ☐ | ☐ |
| Temperaturänderungen | ☐ | ☐ | ☐ | ☐ | ☐ |
| Lärm oder grelles Licht | ☐ | ☐ | ☐ | ☐ | ☐ |
| Stehen / orthostatische Belastungen | ☐ | ☐ | ☐ | ☐ | ☐ |

**PART 2 | Initial Draft Questionnaire (13 items, English translation)**

**Question 1: Which of the following symptoms have you experienced frequently and most of the time since the onset of your illness?**

*Please check all symptoms that apply.*

☐ Post-exertional symptom worsening: After physical, mental, or emotional exertion, a marked worsening of symptoms occurs together with a prolonged recovery time.

☐ Constant exhaustion: Persistent physical and/or mental fatigue that occurs regardless of activity and restricts everyday life.

☐ Muscle pain or burning

☐ Headache or dizziness

☐ Nausea, abdominal pain, or digestive problems

☐ Flu-like symptoms (e.g., sore throat, fever, aching limbs)

☐ Difficulty concentrating (brain fog)

☐ Memory problems

☐ Sensitivity to light

☐ Sensitivity to noise

☐ Circulatory problems or difficulty standing for longer periods

☐ Intolerance or reduced tolerance of certain foods

☐ Unrefreshing sleep or sleep disturbances

☐ Limb or joint pain

☐ Shortness of breath or the feeling of not getting enough air

☐ Palpitations or irregular heartbeat

☐ Sensitivity to temperature changes (e.g., heat/cold)

☐ Low mood or increased irritability

☐ Other symptoms: ____________________________

**Question 2: Compared with your life before the illness, how limited are you in your daily activities?**

☐ 0: Not limited at all

☐ 1: Mildly limited (occasional adjustments needed)

☐ 2: Moderately limited (regular adjustments needed, e.g., breaks)

☐ 3: Severely limited (many activities no longer possible)

☐ 4: Extremely limited (almost all activities are impossible)

**Question 3: Do you experience persistent exhaustion or weakness most of the time that occurs independently of physical, mental, or emotional activity?**

☐ 1. Yes, almost always.

☐ 2. Yes, often, but not constantly.

☐ 3. No, only occasionally.

☐ 4. No, hardly or not at all.

**Question 4: When you are already exhausted, does exertion lead to an additional worsening of symptoms (PEM) that is clearly distinct from your usual exhaustion?**

*Please think about the past 4 weeks.*

☐ 0 = No difference

☐ 1 = Slightly noticeable

☐ 2 = Moderately noticeable

☐ 3 = Clearly noticeable

☐ 4 = Extremely clear difference

**Question 5: After physical, mental, or emotional exertion, do you often experience a disproportionately severe worsening of your symptoms together with a prolonged recovery time?**

☐ 1. Yes, almost always after exertion.

☐ 2. Yes, often, but not always.

☐ 3. Yes, but only rarely.

☐ 4. No, exertion has no additional effects.

**Question 6: During a strenuous activity that is typical for you, how quickly did you feel muscle fatigue? How much did your strength decline?**

*Examples: climbing stairs, repeatedly standing up and sitting down, carrying an object. Past 4 weeks.*

☐ 0 points: No fatigue; the activity remains equally feasible at all times.

☐ 1 point: Occasional mild fatigue, rarely worsening on repetition.

☐ 2 points: Frequently noticeable fatigue, clearly limited with frequent repetition.

☐ 3 points: Very often severe fatigue, with substantial difficulty on almost every repetition.

☐ 4 points: Almost always complete fatigue; the activity can no longer be performed after a few repetitions.

**Question 7: Do you experience worsening of your symptoms after physical activity?**

*Examples: a short walk, climbing stairs, light housework.*

☐ Never (0): I notice no worsening after physical activity.

☐ Rarely (1): I occasionally notice mild worsening.

☐ Sometimes (2): About half the time I notice clear worsening.

☐ Often (3): Worsening occurs frequently after physical activity.

☐ Always (4): Every physical activity leads to clear worsening.

**Question 8: Do you experience worsening of your symptoms after cognitive activity?**

*Examples: reading, focused work at the computer, a longer conversation.*

☐ Never (0): No worsening.

☐ Rarely (1): Occasional mild worsening.

☐ Sometimes (2): Clear worsening about half the time.

☐ Often (3): Frequent worsening.

☐ Always (4): Every cognitive activity leads to worsening.

**Question 9: Do you experience worsening of your symptoms after emotional/mental strain?**

*Examples: stress, arguments, emotional challenges.*

☐ Never (0): No worsening.

☐ Rarely (1): Occasional mild worsening.

☐ Sometimes (2): Clear worsening about half the time.

☐ Often (3): Frequent worsening.

☐ Always (4): Every emotional strain leads to worsening.

**Question 10: Do all of your symptoms worsen after exertion?**

*e.g., physical, cognitive, emotional*

☐ Yes, all symptoms worsen to the same degree.

☐ Yes, all symptoms worsen, but not to the same degree.

☐ No, only some symptoms worsen.

☐ No, only one symptom worsens.

☐ No, no symptom worsens.

**Question 11: How long does the worsened symptom phase usually last?**

☐ A few hours

☐ Less than 24 hours

☐ 24–48 hours

☐ Several days

☐ One week

☐ Several weeks

☐ Does not return to baseline

**Question 12: How well can you recover from the worsening of your symptoms when you rest completely?**

*Please think about the last 4 weeks.*

☐ 1. Very good: I recover completely after a short time (a few hours).

☐ 2. Good: I recover, but it takes at least a day.

☐ 3. Moderate: Recovery is only partial and takes several days.

☐ 4. Poor: There is only minimal improvement, even after prolonged rest.

☐ 5. Not at all: Even after complete rest, my condition improves hardly or not at all.

**Question 13: Are there additional factors that intensify your symptoms after exertion?**

*Please rate each factor separately on a scale from 0 to 4. Last 4 weeks.*

| **Factor** | **0 No effect** | **1 Slight** | **2 Moderate** | **3 Marked** | **4 Extreme** |
| --- | --- | --- | --- | --- | --- |
| Lack of sleep | ☐ | ☐ | ☐ | ☐ | ☐ |
| Stress or emotional strain | ☐ | ☐ | ☐ | ☐ | ☐ |
| Temperature changes | ☐ | ☐ | ☐ | ☐ | ☐ |
| Noise or bright light | ☐ | ☐ | ☐ | ☐ | ☐ |
| Standing / orthostatic load | ☐ | ☐ | ☐ | ☐ | ☐ |
